# SUMOylation-mediated PSME3-20*S* proteasomal degradation of transcription factor CP2c is crucial for cell cycle progression

**DOI:** 10.1126/sciadv.add4969

**Published:** 2023-01-27

**Authors:** Seung Han Son, Min Young Kim, Young Su Lim, Hyeon Cheol Jin, June Ho Shin, Jae Kyu Yi, Sungwoo Choi, Mi Ae Park, Ji Hyung Chae, Ho Chul Kang, Young Jin Lee, Vladimir N. Uversky, Chul Geun Kim

**Affiliations:** ^1^ Department of Life Science and Research Institute for Natural Sciences, College of Natural Sciences, Hanyang University, Seoul 04763, Korea.; ^2^ Department of Molecular Medicine and USF Health Byrd Alzheimer’s Research Institute, Morsani College of Medicine, University of South Florida, Tampa, FL 33612, USA.; ^3^ CGK Biopharma Co. Ltd., Seoul 04763, Korea.

## Abstract

Transcription factor CP2c (also known as TFCP2, α-CP2, LSF, and LBP-1c) is involved in diverse ubiquitous and tissue/stage-specific cellular processes and in human malignancies such as cancer. Despite its importance, many fundamental regulatory mechanisms of CP2c are still unclear. Here, we uncover an unprecedented mechanism of CP2c degradation via a previously unidentified SUMO1/PSME3/20*S* proteasome pathway and its biological meaning. CP2c is SUMOylated in a SUMO1-dependent way, and SUMOylated CP2c is degraded through the ubiquitin-independent PSME3 (also known as REGγ or PA28)/20*S* proteasome system. SUMOylated PSME3 could also interact with CP2c to degrade CP2c via the 20*S* proteasomal pathway. Moreover, precisely timed degradation of CP2c via the SUMO1/PSME3/20*S* proteasome axis is required for accurate progression of the cell cycle. Therefore, we reveal a unique SUMO1-mediated uncanonical 20*S* proteasome degradation mechanism via the SUMO1/PSME3 axis involving mutual SUMO-SIM interaction of CP2c and PSME3, providing previously unidentified mechanistic insights into the roles of dynamic degradation of CP2c in cell cycle progression.

## Teaser

SUMO1/PSME3/20S proteasomal degradation of transcription factor CP2c is crucial for accurate cell cycle progression.

## INTRODUCTION

CP2c (also known as TFCP2, CP2, α-CP2, LSF, LBP-1c, UBP-1, and SEF-1) is a widely expressed member of the *CP2* family of transcription factors (TFs) (*1*–*3*). The *CP2* TF family consists of six isoforms in humans (LBP-1a, LBP-1b, LBP-1c, LBP-1d, LBP-9, and LBP-32, where LBP-1a, LBP-1b, LBP-1c, and LBP-1d are generated by alternative splicing) and four in mice (CP2a/NF2d9, CP2b, CP2c/Tfcp2, and Crtr1/Tfcp2l1, where CP2a and CP2b are generated by alternative splicing) (*1*, *4*, *5*). The genes regulated by CP2c are involved in both tissue-specific functions (including hematopoiesis, immune response, and neural development) (*6*–*10*) and housekeeping functions (like cell proliferation, cell cycle progression, and differentiation) (*11*–*13*). CP2c is important in the pathogenesis of various malignant diseases, such as human immunodeficiency virus infection and acquired immunodeficiency syndrome, allergic response, inflammation, Alzheimer’s disease, and hemoglobinopathies (*14*). CP2c also functions as a protooncogenic factor in various cancers, participates in epithelial-mesenchymal transition and chemoresistance, and enhances angiogenesis (*7*, *13*, *15*–*17*).

The highly divergent spectrum of CP2c actions may be associated with the presence of several specific members of the *TFCP2* family characterized by variations in the DNA binding modules, different interactomes, and specific patterns of tissue distribution (*18*). Although we do not know the underlining molecular mechanisms of how this ubiquitous CP2c exerts such diverse tissue/lineage-specific regulation of gene expression and in human malignancies, some evidence unveiling the molecular mechanisms is emerging. Two kinds of CP2c TF complexes, homotetrameric CP2c complex (tCP2c) and heterohexameric complex (CBP) containing CP2c, CP2b, and Pias1, were suggested (*19*). Recently, we found that a monomeric form of a CP2c homotetramer (tCP2c; [C4]) binds to the known CP2c binding DNA motif [CNRG-N(5~6)-CNRG], whereas a dimeric form of a CP2c, CP2b, and PIAS1 heterohexamer ([C2B2P2]_2_) binds to the three consecutive CP2c half-sites or two staggered CP2c binding motifs, where [C4] exerts a pioneering function for recruiting [C2B2P2]_2_ to the target (*18*). Moreover, whereas all CP2c exists as [C4] or as [C2B2P2]_2_ or [C2B2P2]_4_ in the nucleus, one additional cytosolic heterotetrameric CP2c and CP2a complex ([C2A2]) exerts some homeostatic regulation of the nuclear complexes (*18*).

In addition to CP2c TF complexes by themselves, CP2c often relies on interactions with various partner proteins to regulate the expression of distinct sets of target genes, acting as a transcriptional activator or repressor, including YY1, RING1, RNF2, HDAC1/2, SIN3A, Fe65, NF-E4, TTRAP (TDP2), GATA1, BRD7, and GATAD2A (*8*, *9*, *19*). To gain further insight into the protein-protein interactions that modulate the activities of CP2c, we identified CP2c-interacting proteins using a yeast two-hybrid system (*20*). Among the various putative CP2c-interacting proteins, small ubiquitin-related modifier 1 (SUMO1), along with SUMO-conjugating enzyme (UBE2I) and SUMO ligase (protein inhibitor of activated STAT1, PIAS1), drew our attention as these proteins participate in SUMOylation, which is a fundamental posttranslational modification conserved throughout the eukaryotes, where the target proteins are modified by the covalent conjugation of SUMO to the lysine residue typically positioned within the consensus tetrapeptide motif Ψ-K-x-D/E, with Ψ, K, x, L, D, and E being a hydrophobic residue, lysine, any residue, and aspartate or glutamate (an acidic residue), respectively. This finding raised the question of whether SUMOylation is another way to endow CP2c with regulatory versatility.

SUMO proteins are a family of small proteins that are covalently attached to and detached from other proteins in cells to modify their function and present in five isoforms (SUMO1 to SUMO5) in higher eukaryotes (*21*, *22*). SUMO1 to SUMO3 express ubiquitously, but SUMO4 and SUMO5 are present in limited tissues or cells (*23*–*25*) and have more obscure functions that largely remain enigmatic (*26*). SUMOylation refers to the covalent conjugation of SUMO proteins to the target molecules in a mechanism similar to ubiquitination (*27*–*29*). SUMO2 and SUMO3 (which have 97% peptide sequence similarity and are collectively referred to as SUMO2/3) appeared to carry an internal SUMO consensus motif, enabling the creation of topologically uniform poly-SUMO chains via iterative linkages on K11 (*30*).

SUMO1, which shares about 50% identity with SUMO2 and SUMO3, mainly participates in these chains solely as a terminal cap to preclude further elongation (*31*) and shows distinguishable function, with SUMO2/3 exhibiting different target specificity (*32*, *33*). SUMOylation that involves four enzymatic reactions mediated by SUMO protease and E1, E2, and E3 enzymes is a reaction forming an isopeptide bond between the C-terminal glycine of SUMO and the ε-amino group of a lysine residue in the target protein. This modification could also be highly reversible such that SUMO-specific proteases (SENP1 to SENP7) rapidly cleave SUMOs from target proteins, releasing free SUMO for a new cycle of conjugation (*34*).

SUMO-modified proteins play roles in diverse cellular processes, particularly in transcription, chromatin structure remodeling, and DNA repair, by changing the subcellular localization, altering protein structure, or mediating interactions with other proteins (*35*, *36*). Many of these functions rely on the ability of covalently conjugated SUMO to promote noncovalent protein-protein interactions through a SUMO-interacting motif (*37*) in the interacting protein. SUMO1-interacting motifs (SIMs) generally consist of a four-residue-long hydrophobic stretch of amino acids, with aliphatic nonpolar side chains flanked on one side by negatively charged amino acid residues (*38*–*40*). However, we do not yet fully understand how each SUMOylated protein selects the set of SIM-containing proteins appropriate to its function.

Here, we show that CP2c is SUMOylated in a SUMO1-dependent way and SUMOylated CP2c is degraded through the ubiquitin-independent proteasome activator subunit 3 (PSME3; also known as REGγ, PA28G, or PA28γ)/20*S* proteasome system. In addition, SUMOylated PSME3 could also interact with CP2c to degrade CP2c via the 20*S* proteasomal pathway. Thus, we revealed a unique SUMO1-mediated uncanonical 20*S* proteasome degradation mechanism via the SUMO1/PSME3 axis involving mutual SUMO-SIM interaction of CP2c and PSME3. We also found that the precisely timed degradation of CP2c by this mechanism is required to ensure an accurate progression of the cell cycle.

## RESULTS

### A SUMO1-mediated CP2c SUMOylation system is involved in CP2c degradation

**Fig. 1.**
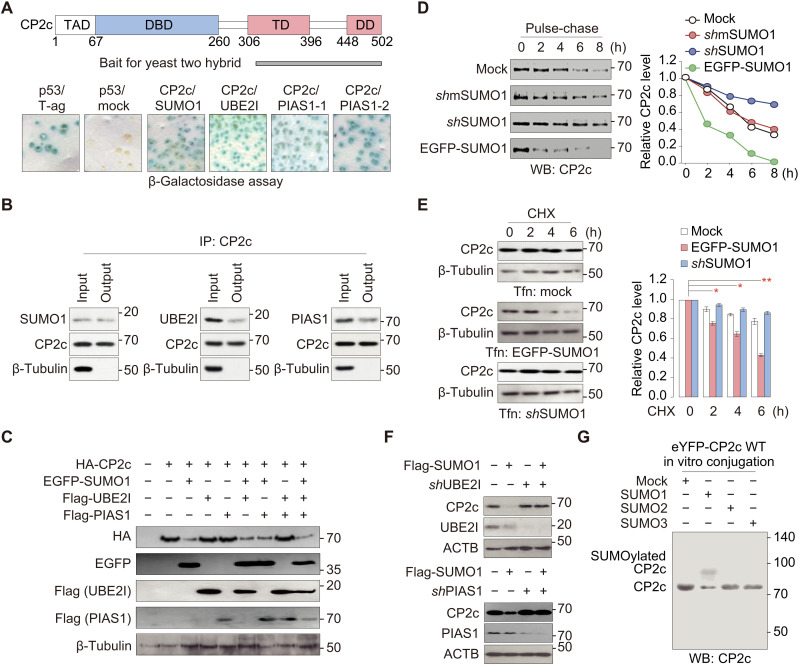
SUMO1, UBE2I, and PIAS1 interact with CP2c and are involved in CP2c degradation probably through CP2c SUMOylation. (**A**) SUMO1, UBE2I, and PIAS1 are identified as CP2c-interacting proteins in the yeast two-hybrid assay. A mouse CP2c protein region used as a bait for yeast two-hybrid is schematically represented along with putative CP2c functional domains (top). The β-galactosidase activities are by colony filter lift assays (bottom). The yeast strain EGY48 was cotransformed with pLexA-CP2c (amino acids 306 to 502) and pB42AD-SUMO1 (1 to 101 amino acids), UBE2I (1 to 158 amino acids), and PIAS1-1 (5 to 651 amino acids). The pLexA-p53/pB42AD-T antigen and pLexA-p53/pB42AD were used as positive and negative controls, respectively. TAD, transcriptional activation domain; DBD, DNA binding domain; TD, tetramerization domain; DD, dimerization domain. (**B**) Representative co-IPs (*n* = 2) showing a direct interaction between CP2c and SUMO1, UBE2I, or PIAS1 in vivo. (**C**) Representative Western blot analyses (*n* = 3) showing CP2c degradation by the SUMOylation system. CP2c is degraded by ectopic SUMO1 expression. (**D** and **E**) SUMO1-mediated CP2c degradation was revealed by measuring CP2c protein half-life with pulse-chase metabolic labeling assays in K562 cells (D) and by measuring the expression level of CP2c protein in 293T cells under the CHX treatment (E). Various SUMO1 constructs were transiently transfected: mock, no transfection; shmSUMO1, mutant short hairpin SUMO1 RNA; shSUMO1, short hairpin SUMO1 RNA; EGFP-SUMO1, EGFP-tagged SUMO1. Data are means ± SD; *n* = 2. *P < 0.05; **P < 0.01. (**F**) Representative Western blot analyses (*n* = 3) show that CP2c degradation by SUMO1 requires UBE2I and PIAS1. (**G**) Representative Western blot (*n* = 2) showing CP2c SUMOylation status by in vitro conjugation assay of SUMO1, SUMO2, or SUMO3. See also fig. S1.

We initially identified SUMOylation machinery proteins SUMO1, UBE2I (Ubc9), and PIAS1 as CP2c interacting proteins by yeast two-hybrid assays using CP2c C-terminal region (306 to 502 amino acids) as a bait. Seven positive clones, including two copies of full-length SUMO1, three copies of full-length UBE2I, and two copies of PIAS1, were isolated (Fig. 1A and fig. S1A). We confirmed CP2c interaction with SUMO1, UBE2I, and PIAS1 by co-immunoprecipitation (co-IP) assays in vitro and in vivo (Fig. 1B and fig. S1B). SUMO1 significantly decreased the transcriptional activity of CP2c by reducing the CP2c protein. When SUMO1 was overexpressed, a ~40% reduction of CP2c’s transcriptional activity was observed (fig. S1C), and the reduction of CP2c’s transcriptional activity was proportional to the SUMO1 expression levels (fig. S1D). CP2c protein levels were largely anticorrelated with the cellular SUMO1 protein levels (Fig. 1C). Moreover, we could also observe SUMO1-mediated CP2c degradation by measuring CP2c protein half-life with pulse-chase metabolic labeling assays in cells (Fig. 1D) or by measuring the expression level of CP2c protein in cells by blocking protein synthesis using cycloheximide (CHX) (Fig. 1E).

However, whereas overexpressed UBE2I or PIAS1 did not alter the effects of SUMO1 on the CP2c transcriptional activity (fig. S1, C and D), PIAS1 knocked down (KD) by short hairpin RNA (shRNA) expression significantly alleviated the suppression effect of SUMO1-mediated CP2c transcription activity (fig. S1E). Moreover, SUMO1-mediated CP2c degradation was also alleviated by arbitral UBE2I or PIAS1 KD (Fig. 1F), suggesting that CP2c degradation requires UBE2I and PIAS1, where the cellular level of UBE2I or PIAS1 is not limiting for CP2c degradation. Because SUMO1, UBE2I, and PIAS1 are involved in protein SUMOylation, we speculated that SUMO1-mediated CP2c degradation occurs via CP2c SUMOylation. SUMO1, but not SUMO2 or SUMO3, is involved in CP2c SUMOylation and degradation in transfected cells (fig. S1F). We also found that CP2c could be SUMOylated in vitro by SUMO1 but not by SUMO2 or SUMO3 (Fig. 1G). These overall data suggest that a SUMOylation system, consisting of SUMO1, UBE2I, and PIAS1, is involved in CP2c degradation.

**Fig. 2.**
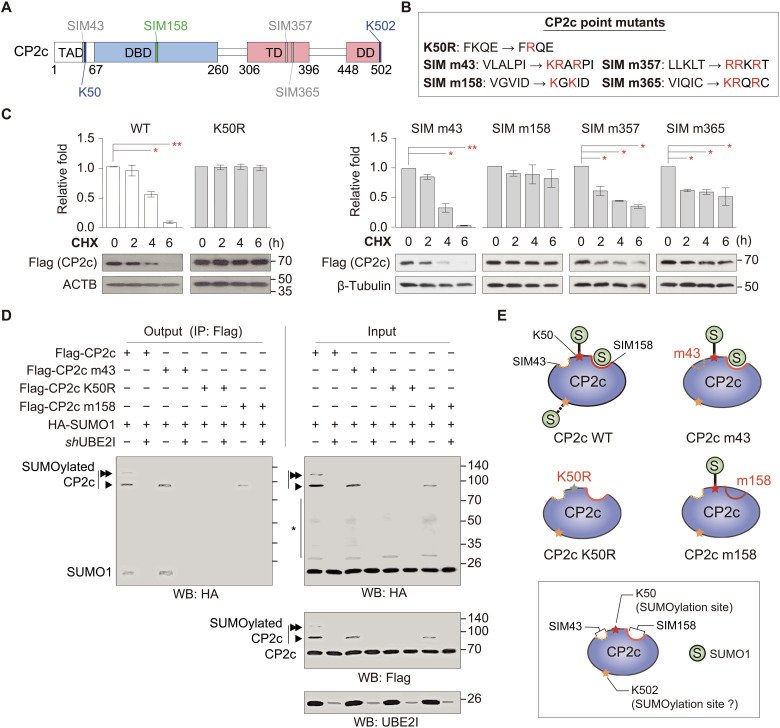
Identification of CP2c SUMOylation- and degradation-related structural signatures in the CP2c protein. (**A**) Schematic drawing of CP2c protein showing putative SUMOylation sites and SIMs predicted by a GPS-SUMO 1.0 program. (**B**) CP2c constructs with point mutations in the putative SUMOylation and SIM sites. (**C**) CP2c protein stability tests of each CP2c mutation. Flag-tagged CP2c WT and various mutants were transiently transfected to 293T cells with HA-SUMO1, and the time-dependent CP2c protein levels were quantified by Western blot. Data are means ± SD; *n* = 2. *P < 0.05; **P < 0.01. (**D**) Representative co-IPs (*n* = 3) showing UBE2I-dependent CP2c SUMOylation phenomenon and the SUMO1 binding and SUMOylation status of each CP2c mutant. It is worth noting that MG132 (50 μM) and NEM (25 mM) were treated in cells for 12 hours before cell harvest and during cell extract preparation, respectively, to enhance SUMOylation signals by preventing CP2c degradation and deSUMOylation. An asterisk indicates other cellular SUMOylated proteins. (**E**) Schematic representation of CP2c SUMOylation sites and SIMs responsible for CP2c SUMOylation and degradation.

To understand the underlying mechanism of how CP2c is degraded by a SUMO1-mediated CP2c SUMOylation system, we started to identify and analyze SUMOylation-related structural signatures in the CP2c protein, i.e., SUMOylation site and SIM. One putative SUMOylated site (K50) and four putative SIMs (SIM43, SIM158, SIM357, and SIM365) were identified using GPS-SUMO 1.0 (Fig. 2A). To evaluate active motifs for CP2c degradation by SUMO1, we constructed *CP2c* variants having a mutation in the SUMOylation site or each of SIMs (Fig. 2B) and subjected to measure the expression level of CP2c protein in cells by transfection of each *CP2c* mutant under the CHX treatment. CP2c degradation was greatly alleviated in K50R- or SIM m158–transfected cells (Fig. 2C), suggesting that K50 and SIM158 are involved in SUMO1-mediated CP2c degradation.

CP2c K50 was confirmed to be a bona fide SUMO1-mediated SUMOylation site by immunoprecipitation (IP) assays (Fig. 2D and fig. S2A), because SUMOylated CP2cs were easily detected in wild-type (WT) CP2c, but not in CP2c mutant K50 (Flag-CP2c K50R)–transfected cells. We could also observe the CP2c K50 SUMOylation in vitro and in vivo by mass spectrometry (MS) (fig. S2, B and C) (*41*). These data suggest that CP2c K50 is responsible for the SUMOylation-dependent CP2c degradation. It is important to note here that, because other TFCP2 isoforms (CP2a and CP2b) have highly conserved amino acid sequences to the CP2c K50 and SIM158 regions, human and mouse CP2a and CP2b are also expected to be SUMOylated and degraded via the SUMOylation-dependent mechanism. However, to simplify the story, we only deal with CP2c degradation and SUMOylation. It is also important to note that the SUMOylated CP2c is prone to degrade in cells, although CP2c SUMOylation occurs via the ordinal SUMOylation machinery, requiring both UBE2I and PIAS1, and thus, it is difficult to detect unless treatments of proteasome inhibitor MG132 and SENP inhibitor *N*-ethylmaleimide (NEM) during the assay is used. Moreover, an additional CP2c SUMOylation could be observed at the C-terminal K502 in MS of samples with ectopic high expression of SUMO1 (see the first lane of Fig. 2D and bottom panel of fig. S2C).

Regarding the alleviation effect of SIM158 mutation (m158) on CP2c degradation (Fig. 2C), it is speculated that SUMO1 binding to CP2c SIM158 is also involved in the SUMOylation-dependent CP2c degradation. A free form of SUMO1 (not SUMOylated) was found to bind to the WT or SIM m43 mutant of CP2c, but not to the K50R or SIM m158 mutant of CP2c (Fig. 2, D and E, and fig. S2A), suggesting that the SUMO1 binding to SIM158 and the K50 SUMOylation should occur concomitantly. In addition, CP2c K50 SUMOylation was metastable in the SIM m158 mutant, suggesting that SUMO1 binding to SIM158 also stabilizes the CP2c K50 SUMOylation status. Together, our data suggest that both SUMO1 binding and SUMOylation are crucial for CP2c degradation, where the concomitant K50 SUMOylation and the SIM158 SUMO1 binding are prerequisites for CP2c degradation (Fig. 2E). It should be noted here that, to cope with this concomitant requirement of K50 SUMOylation and the SIM158 for CP2c degradation, protein-protein interaction though the SUMOylation site and SIM must occur between two molecules of CP2c or between CP2c and other partner proteins (see below).

### The SUMOylated CP2c is degraded through the ubiquitin-independent PSME3/20*S* proteasome system

To reveal the SUMOylated CP2c degradation pathway, we estimated CP2c protein levels in cells treated with different kinds of protease inhibitors (fig. S3A), using MG132 as a proteasome inhibitor and E64 as a lysosomal inhibitor. Treatment with MG132 inhibited SUMO1-induced CP2c degradation, but E64 did not (fig. S3A, lanes 5 to 12), suggesting that the proteasomal degradation pathway is involved in the SUMO1-induced CP2c degradation. However, although most cellular proteins are primarily degraded by the ubiquitin-proteasome pathway, where misfolded proteins are tagged for degradation with a small protein called ubiquitin proteasomes (*42*), the CP2c protein level was not further decreased in cells transfected with His-ubiquitin compared to those of mock or transfected with SUMO1 (fig. S3A, lanes 1 to 4).

Furthermore, nonubiquitinated forms of CP2c with its degradation were only detected in enhanced green fluorescent protein (EGFP)–SUMO1 and hemagglutinin (HA)-ubiquitin transfection, although CP2c could be ubiquitinated by HA-ubiquitin transfection (fig. S3B), suggesting that CP2c degradation occurs mainly through the ubiquitin-independent SUMOylation-proteasomal pathway. Last, CP2c transcriptional activity that was downregulated by SUMO1 was restored in an MG132 treatment-time–dependent manner (fig. S3C), where CP2c protein levels were significantly stabilized (more than twofold), but with no change of *CP2c* transcript levels (fig. S3D). These overall data suggest that SUMO1-induced CP2c degradation occurs at the posttranslational level via ubiquitin-independent and target-specific proteolysis.

**Fig. 3.**
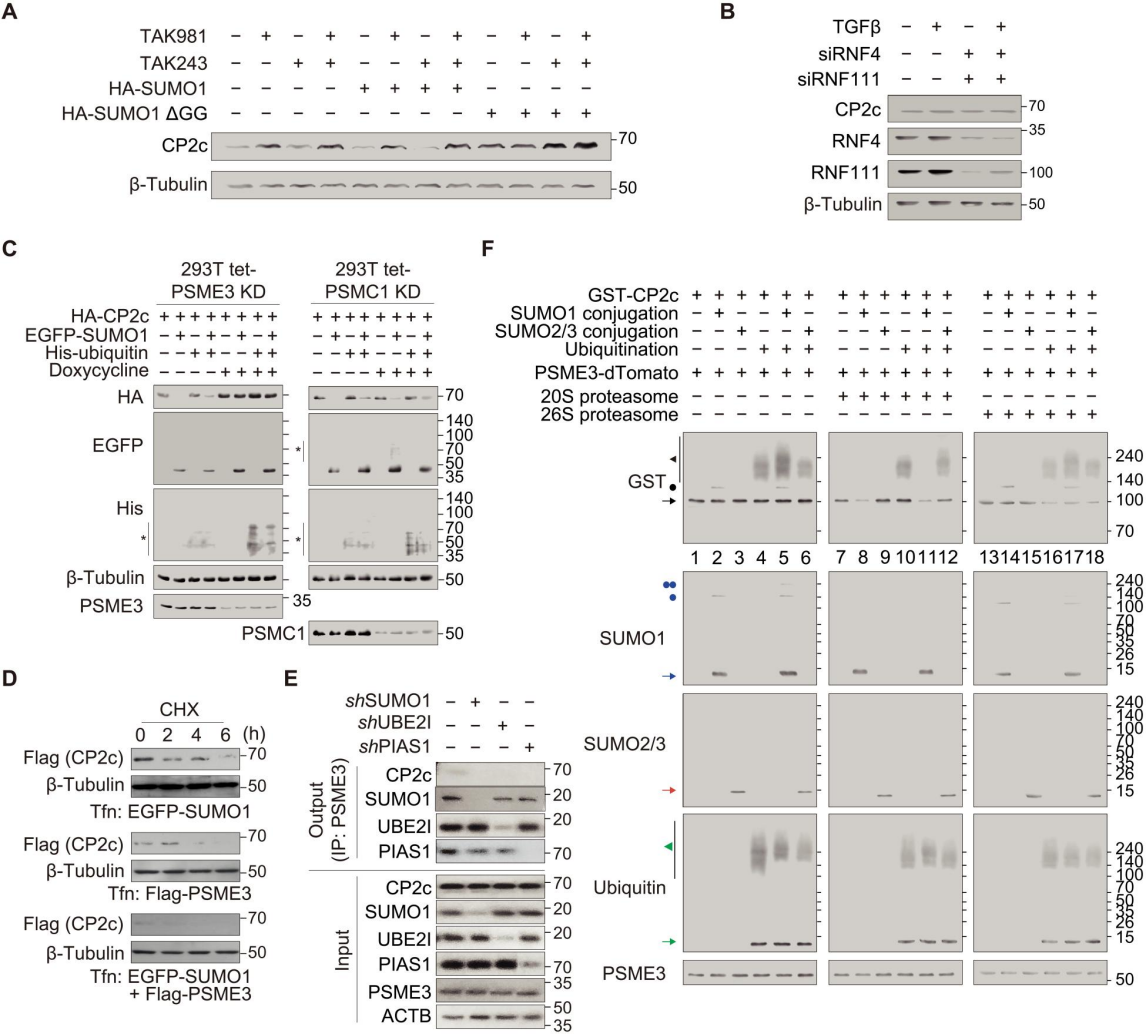
The SUMOylated CP2c is degraded through the ubiquitin- and STUbL-independent PSME3 proteasome system. (**A**) CP2c is degraded via the endogenous SUMO conjugation machinery in a ubiquitin-independent manner in vivo. TAK981 and TAK243, specific inhibitors for the SUMOylation and ubiquitination system, respectively, were used to demonstrate which endogenous system is involved in the degradation of endogenous CP2c in cells. A nonconjugatable SUMO1 form (SUMO1 ∆GG) was also used as a control. *n* = 2. See also fig. S4 for additional data. (**B**) Representative co-IPs (*n* = 3) demonstrate that CP2c degradation occurs in a STUbL-independent manner. Both siRNF4 and siRNF111 were added to the MDA-MB-231 cells to inhibit endogenous STUbLs. TGF-β (1 ng/ml for 6 hours before cell harvest) was added to elicit the RNF111-dependent ubiquitination and degradation condition of poly-SUMOylated substrates, SKI and SKIL. See also fig. S5 for a detailed demonstration of the effects of the RNF4 and RNF111 STUbLs on the degradation of SUMOylated CP2c. (**C**) Representative Western blots (*n* = 2) show that CP2c degradation requires PSME3 (11*S* subunit) (left) but not PSMC1 (19*S* subunit) (right). Doxycycline-inducible PSME3 and PSMC1 KD constructs were transiently transfected with EGFP-SUMO1 or His-ubiquitin or in combination to 293T cells, and the doxycycline-dependent CP2c degradation was monitored. *, other SUMOylated or ubiquitinated proteins. (**D**) Representative Western blots (*n* = 3) showing the markedly reduced CP2c stability in the SUMO1 and PSME3 cotransfected cells. (**E**) Representative co-IPs (*n* = 2) showing binding of PSME3 to endogenous CP2c in UBE2I- and PIAS1-dependent manners. MG132 (10 μM) was treated in cells for 6 hours before cell harvest. (**F**) Representative Western blots (*n* = 2) show that SUMO1-mediated SUMOylation of CP2c is sufficient for degradation by PSME3 (the 20*S* proteasome) in vitro. Colored arrows and dots represent BRCA1- or CP2c-interacted or BRCA1- or CP2c-conjugated factors, respectively.

To confirm that the CP2c degradation occurs in a truly ubiquitin-independent manner, we analyzed the endogenous CP2c degradation in the cells in the presence of a specific inhibitor of the endogenous ubiquitination system (i.e., TAK243; ubiquitin E1 inhibitor) (Fig. 3A and fig. S4, A and C). Here, we also tested whether a specific inhibitor of the endogenous SUMOylation system (i.e., TAK981; SUMO E1 inhibitor) interferes with the degradation of endogenous CP2c (Fig. 3A and fig. S4, B and C). As expected, SUMO1-dependent CP2c degradation was inhibited by TAK981, but not by TAK243, confirming that the ubiquitination system is not involved in the CP2c degradation. Furthermore, overexpression of a nonconjugatable SUMO1 form (SUMO1 ∆GG) abrogated CP2c degradation (Fig. 3A), indicating that the SUMO conjugation machinery rather than the noncovalent SUMO binding is required for the CP2c degradation. We also confirmed that SUMO1, but not SUMO2/3, is involved in SUMOylation and degradation of the endogenous CP2c (fig. S4C).

A literature search revealed no report on the ubiquitin-independent and SUMOylation-dependent proteasomal pathways, although there were studies about SUMOylation-dependent proteasomal pathways through ubiquitination (*43*–*45*). SUMO-targeted ubiquitin ligases (STUbLs), like the mammalian really interesting new gene (RING) finger protein 4 (RNF4) (*46*) and RNF111/Arkadia (*47*), bear multiple SUMO interaction motifs and recognize SUMO polymers to promote target protein degradation via the ubiquitin–26*S* proteasome system by enabling subsequent ubiquitylation (*45*). Although both RNF4 and RNF111 target SUMO polymers of SUMO2/3, RNF111 specifically selects substrates carrying SUMO1-capped SUMO2/3 hybrid conjugates, and its targeting efficiency increases with chain length. However, RNF111 does not recognize a single SUMO1 moiety (*47*). To rule out the possibility of STUbL-mediated CP2c degradation, we tested CP2c SUMOylation and ubiquitination status in the RNF4 and/or RNF111 knockdown MDA-MB-231 cells (Fig. 3B and fig. S5). BRCA1 and SKI/SKIL, positive controls for STUbL-mediated protein degradation via RNF4 and RNF111, respectively, showed increased protein stability after the cell treatment with siRNF4 or siRNF111 (fig. S5) (*41*, *48*). In these experiments, CP2c protein levels were not augmented by siRNF4 and/or siRNF111 treatment (Fig. 3B and fig. S5), confirming that STUbL-mediated protein degradation was not involved in endogenous CP2c degradation.

On the other hand, it is now becoming clear that proteins can be targeted for degradation by the core 20*S* proteasome itself via the ubiquitin-independent pathway (*49*, *50*). This system relies on the intrinsic structural disorder of the protein being degraded (*49*) and is regulated by the association of a 20*S* catalytic subunit with one of the three 11*S* regulatory particles, known as PSME1 to PSME3 (also known as REGα,β,γ or PA28α,β,γ) (*51*). It was shown that PSME1 and PSME2 preferentially form a heteroheptameric immunoproteasome, while PSME3 exists as a homoheptamer, playing a role in a variety of cellular processes (*52*). The 11*S* proteasome does not include any adenosine triphosphatases (ATPases) and can promote the degradation of short peptides, but not of complete proteins.

Structurally abnormal, misfolded, or highly oxidized proteins are also subject to 11*S* proteasome–dependent degradation under conditions of cellular stress (*53*). To discriminate the involvement of 19*S* proteasome from 11*S* proteasome in SUMOylated CP2c degradation, we analyzed CP2c degradation in the conditional KD cells of PSME3 or PSMC1 (a subunit of 19*S* proteasome complex in the 26*S* proteasome pathway) by doxycycline-inducible shRNA expression. We found that SUMO1-induced CP2c degradation was completely suppressed by PSME3 KD but not at all by PSMC1 KD (Fig. 3C). Moreover, PSME3 was more effective for the suppression of CP2c transcriptional activity than SUMO1 by measuring CP2c transcriptional activity with ectopic expression of SUMO1 or PSME3 in cells (fig. S6A). Moreover, the suppression level of CP2c transcriptional activity by both PSME3 and SUMO1 was not different from that of PSME3 alone, suggesting that the SUMO1/PSME3 axis might be involved in CP2c protein degradation-associated suppression of the CP2c transcriptional function. As expected, this SUMO1- and/or PSME3-associated CP2c transcriptional suppression was correlated with the CP2c degradation profiles (Fig. 3D and fig. S6B). We could clearly find the SUMOylation-dependent CP2c-PSME3 interaction, although it was barely seen in MG132-treated cells (Fig. 3E). We could show with a fully recombinant system that SUMO1-mediated SUMOylation of CP2c is sufficient for degradation by the 20*S* proteasome, although CP2c is degradable a bit by the ubiquitination system (Fig. 3F). The overall findings indicate that the PSME3/20S proteasome pathway is involved in SUMOylated CP2c degradation.

### CP2c and PSME3 recognize each other through mutual SUMO-SIM interactions

**Fig. 4.**
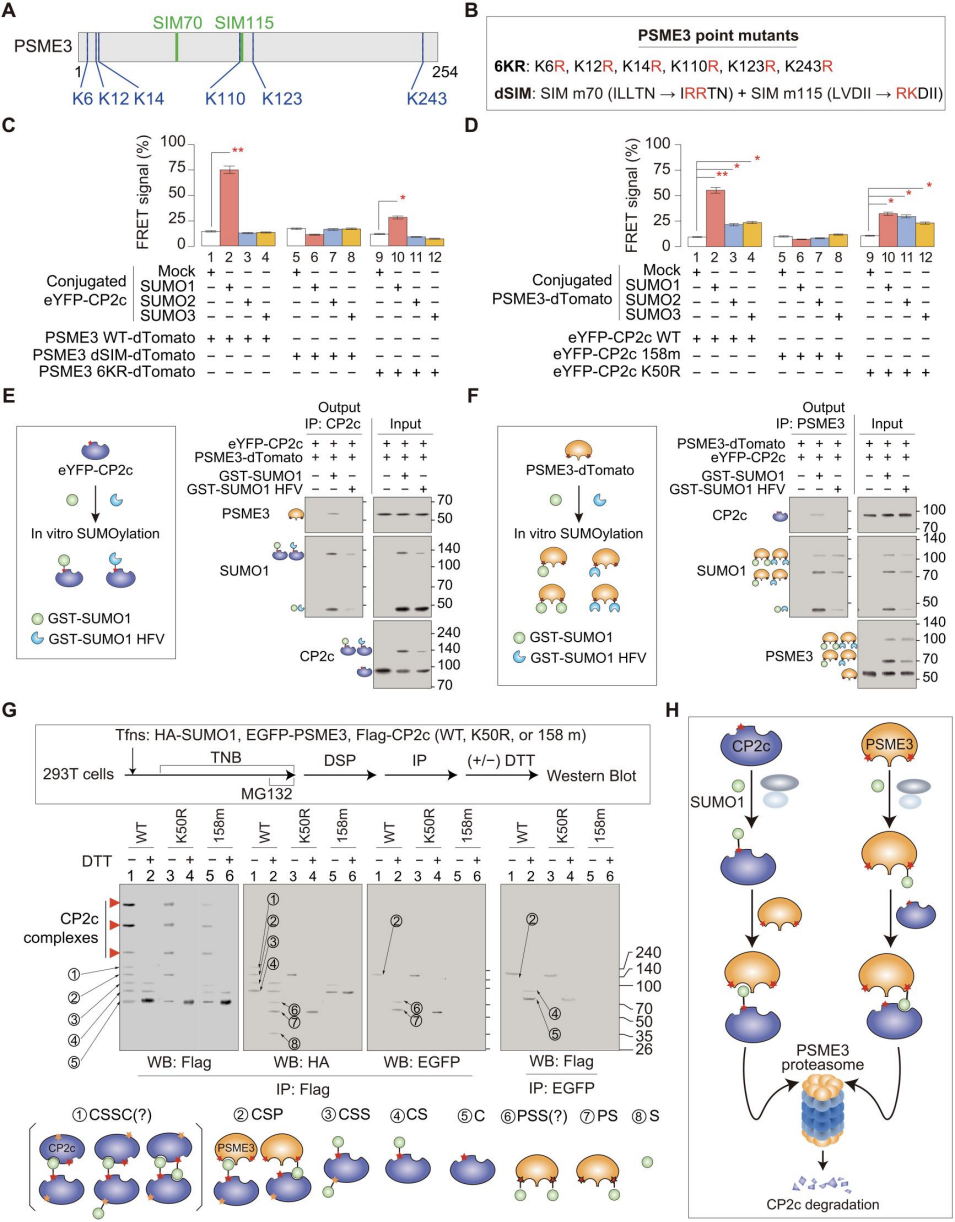
CP2c and PSME3 recognize each other through mutual SUMO-SIM interactions. (**A**) Schematic representation of PSME3 showing putative SUMOylation and SIM sites. (**B**) Various PSME3 mutant constructs with point mutations in the putative SUMOylation and SIM sites. (**C** and **D**) Relative mean FRET percentages between the eYFP-CP2c WT with or without SUMO conjugation and the non-SUMOylated PSME3 WT, dSIM, or 6KR-dTomato, respectively (C), and between the PSME WT-dTomato with or without SUMO conjugation and the non-SUMOylated eYFP-CP2c WT, 158m, or K50R, respectively (D). *n* = 3. See fig. S7 (A to F) for the original data. (**E** and **F**) Representative co-IP assays (*n* = 2) show that CP2c and PSME3 recognize each other through the direct CP2c-SUMO1-PSME3 interaction. Epitope-tagged CP2c (E) or PSME3 (F) were subjected to the in vitro SUMOylation reaction using WT or the SUMO1 HFV mutant, and the resulting samples were incubated with PSME3 or CP2c, respectively, to see specific interactions using co-IPs. (**G**) Representative DSP XL-IP analyses (*n* = 3) identifying PSME3-CP2c protein subcomplexes in the reactions containing WT or CP2c mutants. The schematic experimental procedure (top) and the expected protein complex models (marked by the circled letters in the Western blots; bottom) are depicted. DSP, dithiobis (succinimidyl propionate); DTT, dithiothreitol; FRET, fluorescence resonance energy transfer; TNB, thymidine-nocodazole block; XL-IP, crosslinking immunoprecipitation. (**H**) Schematic model showing CP2c degradation through two ubiquitin-independent PSME3 proteasome pathways. CP2c degradation could occur by the coupled interactions of CP2c and PSME3 through bindings between SUMO in one protein and SIM in the other protein.

Because CP2c degradation through a PSME3/20*S* proteasome pathway requires both CP2c SUMOylation at K50 and SIM158 (Fig. 2E), and PSME3 is known to be SUMOylated by SUMOs (*54*), we could simply imagine that both CP2c SUMOylation site K50 and SIM158 correspondingly interact with PSME3 SIM(s) and the SUMOylation site(s) for the degradation of SUMOylated CP2c. GPS-SUMO 1.0 analyses identified multiple SUMOylated sites, including K6, K12, and K14, and two SIMs (SIM70 and SIM115) in PSME3 (Fig. 4A). We tested whether PSME3 SUMOylation is involved in the interaction with CP2c SIM158 for CP2c degradation using PSME3 mutants of the putative SUMOylation sites (PSME3 6KR, having mutations in all six putative SUMOylation sites) and SIMs (PSME3 dSIM, having mutations in putative SIM70 and SIM115) (Fig. 4B). In accordance with the previous report (*54*), PSME3 could be SUMOylated in vitro by SUMO1, SUMO2, and SUMO3 (fig. S7A). CP2c degradation was not induced in the PSME3 6KR mutant (fig. S7B), demonstrating that SUMOylated PSME3 is really involved in the CP2c degradation process.

In addition, we found direct binding of SUMOylated CP2c to PSME3 SIMs and SUMOylated PSME3 to CP2c SIM158 in vitro by fluorescence resonance energy transfer (FRET) assays (Fig. 4, C and D, and fig. S7, C to F) and Western blots (fig. S7, G and H). It is important to note here that the CP2c binding ability of the SUMO1-conjugated PSME3 is greatly reduced by the CP2c K50 SUMOylation mutant (compare lane 10 with lane 2 in Fig. 4D), recalling the previous notion that SUMO1 binding to SIM158 also stabilizes the CP2c K50 SUMOylation status (Fig. 2, D and E). SUMO2- or SUMO3-conjugated PSME3 could also interact with CP2c, where they showed the effective binding capability to the SUMOylation-defective CP2c (K50R mutant) when compared with the SUMO1-conjugated PSME3, although their binding capability to the WT CP2c was less effective (compare lanes 2 to 4 with lanes 10 to 12 in Fig. 4D). These findings indicate that CP2c is degraded by 20*S* proteasome when either the SUMOylated CP2c interacts with the PSME3 SIMs or the SUMOylated PSME3 interacts with the CP2c SIM, showing some synergism to induce mutual interaction between them.

To further validate the SUMO1-SIM–dependent binding of CP2c and PSME3, we used a SUMO1 HFV (H35A/F36A/V38A) mutant, a SUMO1 variant that is defective in SIM binding (*55*). As expected, a SUMO1 HFV mutant did not invoke CP2c degradation in the cell-based assays (fig. S7I), as SUMOylated CP2c and PSME3 by SUMO1 HFV did not interact with PSME3 or CP2c, respectively, irrespective of its SUMOylation status (fig. S7, J and K). When we performed in vitro reconstituted binding studies, in contrast to the SUMOylated CP2c and PSME3 by WT SUMO1, both SUMOylated CP2c and PSME3 by SUMO1 HFV did not interact at all with PSME3 and CP2c, respectively (Fig. 4, E and F). These findings confirm that the SUMO1-mediated SUMOylation of CP2c and PSME3 is involved in the interaction with the corresponding partner PSME3 or CP2c SIMs, respectively, to induce CP2c degradation.

To test whether the specific interactions between CP2c and PSME3 really occur through SUMO-SIM, we also performed dithiobis (succinimidyl propionate) (DSP) crosslinking and immunoprecipitation (DSP XL-IP) analysis in cells transfected with WT or mutant CP2c in the K50 SUMOylation site or SIM158 (Fig. 4G, top). Because DSP is a disulfide bond–containing crosslinking reagent and can be chemically cleaved by reducing reagent treatment, DSP XL-IP could identify components in the crosslinked complex and estimate their stoichiometry. However, it is of note that, because crosslinked protein complexes do not migrate according to molecular weight standards and the molecular weight standards are only in the range of 50 to 240 kDa, our estimation of the mass of crosslinked proteins by determining the relative migration distance of the protein standards may have inherent caveats. Nevertheless, we could distinguish several CP2c-PSME3 subcomplexes formed by DSP crosslinking and/or SUMOylation.

When comparing the nature of crosslinked subcomplexes and the SUMOylation status of CP2c and PSME3, specific interactions between CP2c and PSME3 were impaired by CP2c K50R or SIM158m mutation. Further validation of interactions between the CP2c SUMO and PSME3 SIM(s) and between the PSME3 SUMO and CP2c SIM was obtained by MS in vitro and in vivo, providing structural models about specific interactions between SUMO and SIMs (fig. S8). CP2c SUMOylation was not observed in the three kinds of nuclear CP2c transcription complexes (namely, [C4], [C2B2P2]_2_, and [C2B2P2]_4_) (*18*), although the amounts of these complexes were reduced by CP2c mutation in K50 or SIM158 (Fig. 4G, bottom), suggesting that either CP2c SUMOylation occurring in the CP2c complexes rapidly induces complex dissociation or CP2c SUMOylation does not occur in CP2c within the complex.

In addition, the crosslinked CP2c dimer (denoted by band ① in Fig. 4G) was observed in CP2c WT, but not in the K50 or SIM158 mutant, suggesting that the intact SUMO-SIM interaction between two CP2cs is required for the formation of stable CP2c dimer. Band ⑥ was observed in CP2c WT, but not in the K50 or SIM158 mutant, and based on the project molecular weight is expected to have one PSME3 with two SUMOs, suggesting that two SUMO1s might be simultaneously conjugated to the six putative SUMOylation sites of PSME3, where either one of the SUMOylation sites is engaged in binding to CP2c SIM. Together, our data indicate that CP2c and PSME3 recognize each other via interactions between K50 SUMOylated CP2c and PSME3 SIMs and between SUMOylated PSME3s and CP2c SIM158 to induce CP2c degradation by 20*S* proteasome (Fig. 4H).

### SUMO1/PSME3/20*S* proteasome axis–mediated CP2c degradation is required for proper cell cycle progression

As we witnessed, the steady-state CP2c protein level was greatly reduced in cell extracts prepared in the absence of protease inhibitor MG132, and the SUMOylated CP2 was not easy to see in cell extracts prepared in the absence of MG132 and SENP inhibitor NEM (Fig. 2D and fig. S4C). Furthermore, about 25% of nuclear CP2c was in the process of degradation (i.e., SUMOylated or complexed with PSME3 via mutual SUMO-SIM interaction), when cells were arrested at the G_2_-M phase in the presence of MG132 (Fig. 4G). Because all CP2c was found in CP2c transcription complexes [C4], [C2B2P2]_2_, and [C2B2P2]_4_, with no degradation intermediate or free form of CP2c in the nuclear extracts prepared in the absence of MG132 (*18*), we speculate that high levels of CP2c might be degraded through the SUMO1/PSME3/20*S* proteasome axis in cells, raising the question of why CP2c should degrade so highly in the cells, and when it occurs during regular cell cycle progression.

The PSME3/20*S* proteasome is known as an important pathway in multiple biological processes, including cell growth, cell cycle regulation, and apoptosis, by promoting the degradation of the cell cycle regulatory proteins like p21(WAF/CIP1), p16(INK4A), and p19(ARF), and oncogene SRC3 in adenosine triphosphate (ATP)– and ubiquitin-independent manner (*56*, *57*). PSME3, which is largely confined to the nucleus (*58*), was revealed to show cytosolic translocation by SUMOylation, causing increased stability of this proteasome activator to enhance degradation of the cell cycle regulator p21 (*54*). Meanwhile, CP2c has been known as a regulator in the progression from the G_1_ to the S phase of the cell cycle by inducing thymidylate synthase, promoting DNA synthesis, and functioning as an antiapoptotic factor (*12*). Moreover, CP2c functions as an oncogene in hepatocellular carcinoma, showing a positive correlation of CP2c expression levels to the progression stage of tumors (*17*).

Accordingly, we hypothesized that CP2c should also degrade timely and massively after the completion of oncogenic transcription function in the S phase, as timely up-regulation of CP2c might be required for the G_1_ to S transition. SUMOylated CP2c is expected to be degraded by the PSME3/20*S* proteasome pathway in the nuclear periphery because RanGAP1, the major cellular reservoir for the limited amount of SUMO1 and from where the modifier can be dynamically mobilized to be conjugated onto other targets as needed, localizes around the nuclear pore complex (*26*), whereas most of the PSME3/20*S* proteasome exists in the nucleus (*59*, *60*). It is also expected, however, that SUMOylated CP2c could also be degraded in the cytosol by PSME3/20*S* proteasome as nuclear membrane breakdown at the M phase. On the contrary, because SUMOylated PSME3 appears to translocate into the cytosol (*54*), SUMOylated PSME3 could also degrade CP2c in the cytosol through the PSME3/20*S* proteasome. Therefore, it is speculated that the timely and massive CP2c degradation through the SUMO1/PSME3/20*S* proteasome pathway has biological meaning in maintaining proper levels of CP2c during cell cycle progression.

**Fig. 5.**
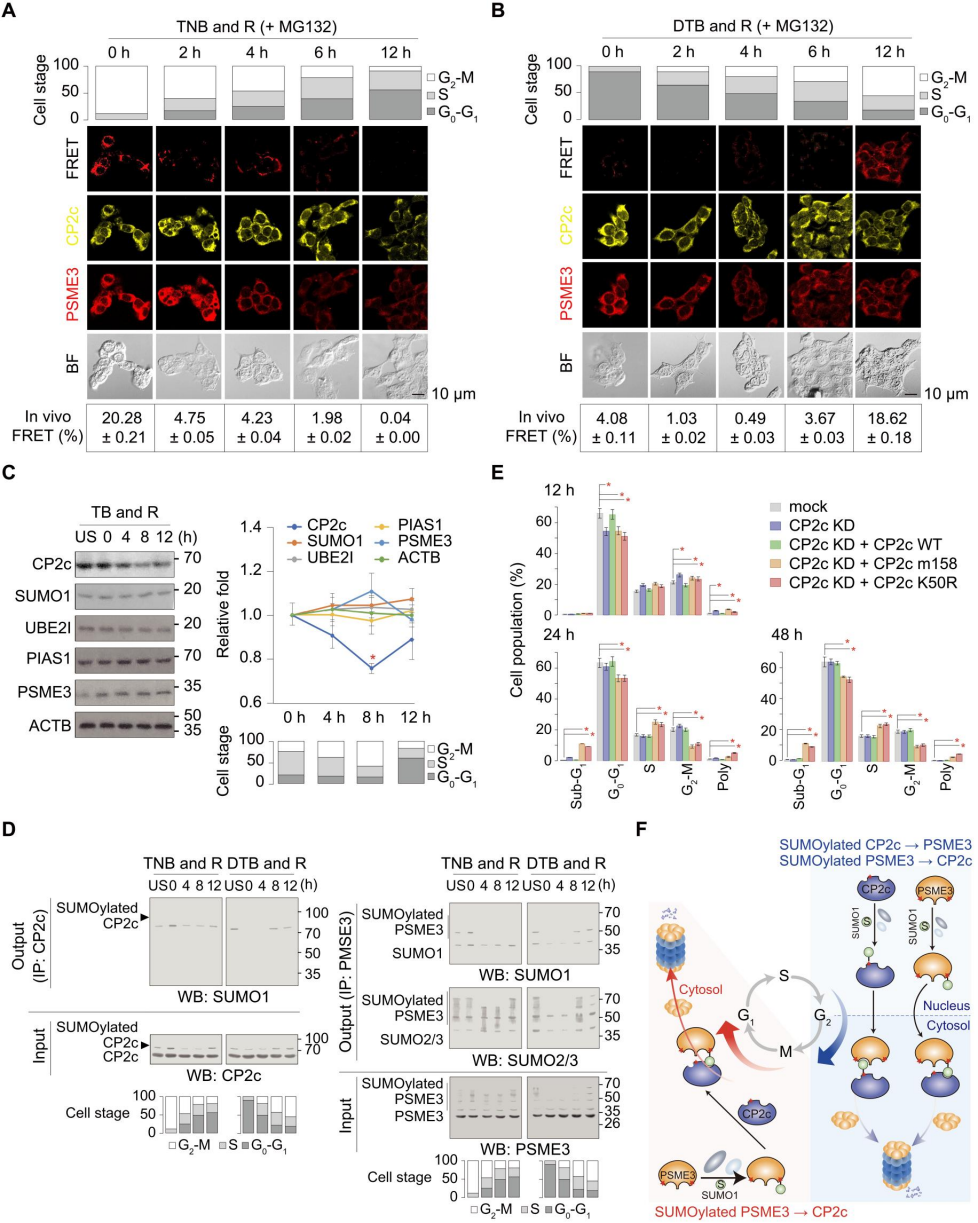
CP2c degradation through the SUMO1/PSME3/20*S* proteasome axis is crucial for proper cell cycle progression. (**A** and **B**) Confocal microscopy showing the CP2c colocalization with PSME3 at the G_2_-M phase of the cell cycle. The CP2c eYFP-tagged CP2c and the dTomato-tagged PSME3 were transfected into 293T cells, and the synchronized cells were prepared by the TNB & R protocol (A) or by the DTB & R protocol (B). FRET signals were considered as colocalization. Data are means ± SD; *n* = 2. (**C**) Representative Western blots of proteins (left) and the quantified expression (right) in 293T cells after a thymidine block and release (TB & R). Data are means ± SD of duplicated experiments; *P < 0.05. The percentage of cell populations is shown at the bottom. (**D**) Representative co-IPs (*n* = 2) showing a cell cycle stage–dependent distribution of SUMOylated CP2c and PSME3 in the CP2c-PSME3 complexes. See also fig. S9 for additional data. (**E**) Cell cycle distribution profiles show the effects of the CP2c mutation in either SUMOylation or SIM on cell cycle progression. *n* = 2, *P < 0.05. See fig. S11 (A and B) for the overlay histograms of the original cell cycle analyses and similar analyses using CP2c dominant negative (*62*). (**F**) Schematic depiction of two CP2c degradation mechanisms during cell cycle progression. Red-colored asterisks represent SUMOylation sites, whereas grooves in the protein represent SIMs.

We set up several different experiments to test our hypothesis. First, to see the cell cycle–dependent colocalization of CP2c and PSME3, we chased the FRET signals at different time points after release from the G_1_-S or G_2_-M stage synchronization in 293T cells, where the CP2c enhanced yellow fluorescent protein (eYFP)–tagged CP2c and the dTomato-tagged PSME3 were transfected, by confocal microscopy (Fig. 5, A and B). MG132 and NEM were treated in cells for 6 hours before releasing the cell cycle arrest and during cell extract preparation, respectively, to enhance the FRET signals by preventing CP2c degradation.

When we scored the percentage of cell populations in each phase of the cell cycle and quantified FRET signals in confocal images, FRET signals were maximal in the G_2_-M phase in both experiments, culminating up to 20% of cells (Fig. 5, A and B). The endogenous CP2c and PSME3 also showed a similar percentage of colocalization signals in the cytosol at the G_2_-M phase of the cell cycle as revealed by immunocytochemistry/confocal microscopy of MDA-MB-231 and 293T cells prepared by the same TNB & R and DTB & R protocols (fig. S9, A to D). Next, we found that CP2c degradation started at the late S phase and was maximal at the G_2_-M phase when we analyzed the CP2c protein levels in the cells obtained using the TB & R protocol (without MG132 treatment) by Western blots (Fig. 5C) and in cells obtained by using the TNB & R and DTB & R protocols (without MG132 treatment) by immunocytochemistry/confocal microscopy (fig. S9, E to H). Therefore, the timely and massive CP2c degradation through the SUMO1/PSME3/20*S* proteasome pathway mainly correlates with the nuclear envelope breakdown in the mitotic cells. Because of this nuclear envelope breakdown in mitotic cells, there is no possibility to distinguish between the cytoplasm and nucleus anymore, and therefore, SUMOylated CP2c and PSME3 could easily meet with SIMs of their partners, PSME3 and CP2c, to degrade CP2c protein. Furthermore, a small fraction (1 to 3%) of cellular SUMO1 was identified in the cytosol of G_1_-S-G_2_ phase cells with a clear nuclear envelope (fig. S10). Our data are consistent with the previous reports that PSME3 SUMOylation mediates cytosolic translocation and increases the stability of PSME3 (*54*, *61*), and suggest that cytosolic CP2c could also be degraded by SUMOylated PSME3 in the cytosol. Therefore, most CP2c colocalizes with SUMO1 and PSME3 in the cytosol of cells to be degraded by 20*S* proteasome at G_2_-M-G_1_.

Last, when the cell cycle–dependent SUMOylation profiles of CP2c and PSME3 were analyzed in cell cycle–synchronized cells by co-IPs, CP2c SUMOylation appeared in most of the cell stages with maximal at the G_2_-M stage, whereas the SUMO1-conjugated PSME3 SUMOylation appeared at the G_2_-M stage, while the SUMO2/3-conjugated PSME3 SUMOylation appeared in most cell stages with different SUMOylation profiles in a cell stage–dependent manner (Fig. 5D). Together, these data suggested that the SUMO1/PSME3/20*S* proteasomal degradation of CP2c occurs starting from the late S phase until G_0_-G_1_ phase through G_2_-M, with a maximum at the G_2_-M phase.

To demonstrate whether CP2c degradation through the SUMO1/PSME3/20*S* proteasome axis is critical for proper cell cycle progression, we analyzed the CP2c SIM or SUMOylation mutation effect on cell cycle progression in 293T cells, in which endogenous CP2c expression was suppressed by transfection of the CP2c shRNA (Fig. 5E and fig. S11A) or the CP2c dominant negative (fig. S11B) (*62*), by flow cytometry. We found that the CP2c SIM- or SUMOylation-mutant cell line showed abnormal cell cycle progression at each phase with increased polyploidy and cell death (sub-G_1_ phase cells) (Fig. 5E and fig. S11). These results suggested that proper spatiotemporal degradation of CP2c is required for normal cell cycle progression. Consequently, we propose two different CP2c degradation mechanisms for proper cell cycle progression (Fig. 5F). CP2c is SUMOylated by SUMO1 during the S to the G_2_-M phase transition, and the SUMOylated CP2c binds to PSME3 in the nuclear envelope to induce CP2c degradation via 20*S* proteasome at the G_2_ phase. As the nuclear membrane breaks down at the M phase, the SUMOylated CP2c could also degrade in the cytoplasm. On the other hand, during the M to the G_0_-G_1_ phase transition, CP2c binds to the cytosolic SUMOylated PSME3, resulting in CP2c degradation in the cytosol.

### The SUMO1/PSME3/20*S* proteasome system is also involved in the cell cycle–dependent degradation of some other nuclear factors

**Fig. 6.**
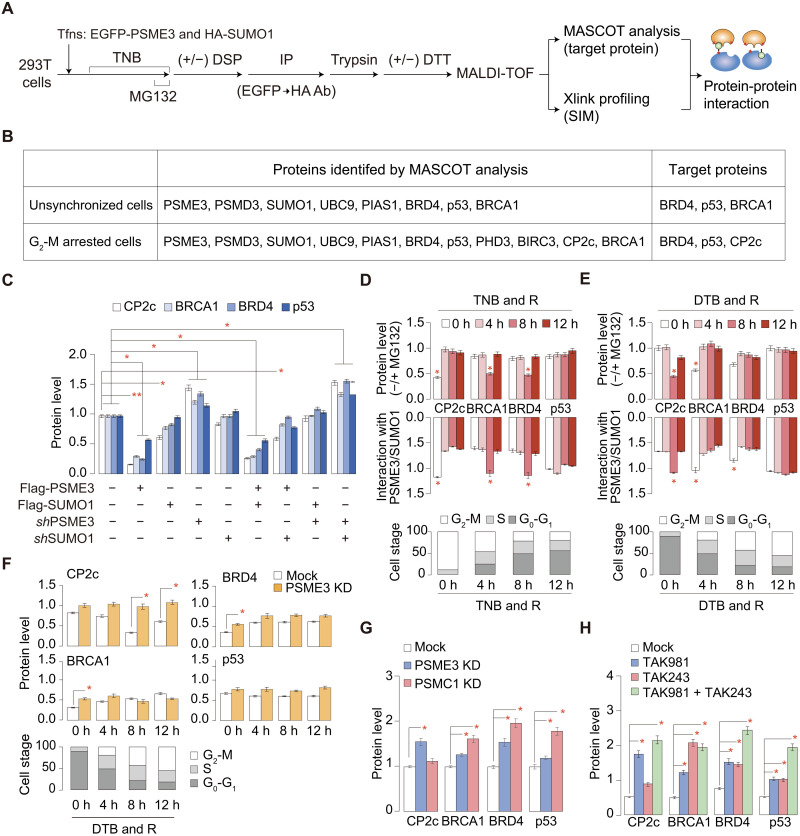
The SUMO1/PSME3/20S proteasome system is also involved in the cell cycle–dependent degradation of some other nuclear factors. (**A** and **B**) Experimental scheme identifying the cellular proteins simultaneously interacting with both SUMO1 and PSME3 (A) and the identified proteins (B). A protein was scored as positive when it appeared repeatedly in duplicated experiments. See also fig. S12A. (**C**) Western blot testing the SUMO1/PSME3 proteasome–dependent protein degradation in 293T cells, where various constructs were transiently transfected. See also fig. S12B. (**D** and **E**) Cell stage–dependent protein levels and protein interactions with SUMO1 and PSME3 were measured in cells obtained by a TNB & R (D) or a DTB & R (E) protocol in the presence or absence of MG132 (see also fig. S12, C and D). (**F**) Cell stage–specific protein degradation profiles in cell stage–synchronized PSME3 KD cells (see also fig. S12E). (**G** and **H**) Tests discriminating two mechanisms of protein degradation, a ubiquitin-dependent 26*S* proteasome pathway, and a SUMO1/PSME3 proteasome pathway. Cellular protein levels were quantified in PSME3 or PSMC1 KD 293T cells (G) or in cells where SUMOylation and/or ubiquitination were inhibited (H) (see also fig. S12, E and F). All of the data in each panel obtained from duplicated experiments are shown as means ± SD. *P < 0.05; **P < 0.01.

To our knowledge, this study is the first report describing the SUMO1/PSME3/20*S* proteasome axis–mediated protein degradation, although PSME3/20*S* proteasome–mediated protein degradation (*49*) or SUMOylated PSME3-mediated protein degradation (*54*, *63*) was reported. We expect that there might exist additional proteins to be degraded through the SUMO1/PSME3/20*S* proteasome axis if this protein degradation mechanism really functioned in eukaryotic cells. Accordingly, we attempted to search for proteins that concomitantly interact with SUMO1 and PSME3 using the DSP crosslinking sequential IP-MS protocol (Fig. 6A). The MASCOT analyses revealed BRD4, p53, BRCA1, and CP2c as target proteins simultaneously interacting with both SUMO1 and PSME3 (Fig. 6B). When we analyzed CP2c peptide fragments crosslinked to the SUMOylated PSME3 peptide fragments, we could identify CP2c SIM158 as a putative target for interaction with the PSME3 SUMO (fig. S12A).

In a similar analysis, we could identify putative SIMs in all of the selected target proteins (fig. S12A), suggesting that these selected proteins really interact with PSME3 via SUMOylation. Moreover, as revealed by Western blots of target proteins in cells with various modulations of PSME3 and SUMO1 protein levels, we found that all target proteins showed PSME3- and SUMO1-dependent degradation, although the degradation levels were somewhat different among target proteins (Fig. 6C and fig. S12B). Moreover, all target proteins showed cell cycle–specific degradation through interaction with PSME3 and SUMO1—while CP2c is degraded at the G_2_-M phase, BRCA1 and BRD4 are degraded at the G_0_-G_1_ phase, and p53 is degraded at all stages of the cell cycle (Fig. 6, D and E, and fig. S12, C and D).

Moreover, by analyzing protein degradation profiles in doxycycline-induced PSME3 or PSMC1 knockdown cells, we found that all target proteins could be degraded through both 26*S* and SUMO1/PSME3/20*S* proteasome pathways—regardless of the major degradation pathways, the SUMO1/PSME3/20*S* proteasome pathway is involved in a cell cycle–specific target protein degradation in all cases (Fig. 6F and fig. S12E). CP2c degradation occurred mainly through the SUMO1/PSME3/20*S* proteasome pathway, degradation of BRCA1 and BRD4 was equally through the 26*S* and SUMO1/PSME3/20*S* proteasome pathways, and p53 degradation occurred mainly through the 26*S* proteasome pathway (Fig. 6G and fig. S12E). We validated protein degradation of these nuclear factors through both 26*S*, probably through the STUbL pathway, and SUMO1/PSME3/20*S* proteasome pathways in endogenous cells by using specific inhibitors of SUMOylation and/or ubiquitination (Fig. 6H and fig. S12, F and G). Together, a protein degradation system through the SUMO1/PSME3/20*S* proteasome axis functions in cells to degrade some nuclear proteins, including CP2c, BRCA1, BRD4, and p53, in a cell cycle–specific manner.

## DISCUSSION

In this study, we demonstrated that an unforeseen SUMO1/PSME3/20*S* proteasome pathway is involved in a cell cycle–specific CP2c degradation via mutual SUMO-SIM interactions of CP2c and PSME3—the SUMO1-conjugated CP2c interacts with PSME3 SIMs to be degraded ubiquitin-independently via the 20*S* proteasomal pathway, while SUMOylated PSME3 could also interact with CP2c SIM158 to degrade CP2c. Moreover, the precisely timed degradation of CP2c by the SUMO1/PSME3/20*S* proteasome axis is required to ensure an accurate progression of the cell cycle. Therefore, our results highlight the identification of a unique SUMO1-mediated uncanonical PSME3/20*S* proteasome degradation mechanism and provide previously unidentified mechanistic insights into the roles of dynamic degradation of CP2c in cell cycle progression with mutual SUMO-SIM interactions of CP2c and PSME3.

In this study, we found that CP2c is SUMOylated at K50 through the SUMO1-mediated system, but not through the SUMO2/3-mediated system, in vivo and in vitro. In our in vitro SUMOylation analysis, we could easily see SUMOylated PSME3 and BRCA1, but not CP2c, by SUMO2 and SUMO3 (Figs. 1G and 3F and fig. S7A). We also could not see any endogenous or native SUMOylated CP2c by SUMO2/3 even in the presence of proteasome inhibitor MG132 and SENP inhibitor NEM, where SUMO1-mediated SUMOylated CP2c was easily seen (figs. S4 and S5). However, Hendriks *et al.* (*64*) reported that the endogenous and natural CP2c could be SUMOylated at K50 by SUMO2/3. Although SUMO2/3 system–mediated CP2c SUMOylation did not significantly affect the CP2c degradation, whether CP2c could be SUMOylated by SUMO2/3 is controversial. One explanation would be that SUMO2/3-mediated CP2c SUMOylation could occur but would be vulnerable to proteolysis and/or deSUMOylation during the handling of samples.

Here, our results show another ubiquitin-independent, 20*S* proteasome–involved proteolytic pathway, which corresponds to a unique SUMO1-mediated uncanonical PSME3/20S proteasome degradation mechanism (i.e., a SUMO1/PSME3/20*S* proteasome pathway), involving mutual SUMO-SIM interactions in between PSME3 and the protein target to be degraded by the 20*S* proteasome. This SUMO1/PSME3/20*S* proteasome–involved proteolytic pathway has been known to be implicated in viral infection and replication. For example, the host SUMO system (SUMO1/UBC9/PIAS2) is required for hepatitis C virus (HCV) replication by ubiquitin-independent degradation of the HCV core proteins, NS3 and NS5A, through the PSME3 SUMOylation–dependent proteasome pathway (*65*–*67*). In addition, during coxsackieviral infection, the host SUMO1/PSME3/20*S* proteasome system induces a redistribution of PSME3 from the nucleus to the cytoplasm to degrade p53 via 20*S* proteasome in the cytosol, rendering viral replication (*63*).

Furthermore, SUMOylation-deficient PSME3 had reduced activity in p21 degradation (*54*), suggesting that the SUMO1/PSME3/20*S* proteasome is involved in eukaryotic cellular protein degradation. Because the loss of the SUMOylation pathway is known to impair cell cycle progression at the G_1_ stage and cancer proliferation by targeting other cell cycle regulators (*68*, *69*), the SUMO1/PSME3/20*S* proteasome axis–dependent protein degradation system is expected to be broadly involved in the regulation of cell cycle progression. Last, we found that other nuclear factors, like BRD4, BRCA1, and p53, could be additional targets of this SUMO1/PSME3/20*S* proteasome axis–dependent protein degradation system, although their preference for protein degradation via the PSME3/20*S* proteasome or 26*S* proteasome pathway (possibly in a STUbL-dependent manner) was variable among them (Fig. 6 and fig. S12). Therefore, the SUMO1/PSME3/20*S* pathway is speculated to function as another major cellular function of protein degradation, although further studies are required for the elucidation of the detailed molecular mechanisms and cellular meaning.

Protein homeostasis is a process that controls the amount of cellular protein to ensure normal cellular dynamics, but the exact mechanism of proteolysis through posttranslational modification such as SUMOylation is still not fully understood. Although both SUMOylation and ubiquitination are important reversible posttranslational modifications that occur at Lys residues, the key difference between them is that ubiquitination can mark proteins for proteolytic degradation via proteasomes or have other signaling functions, whereas SUMOylation is not used to mark proteins for degradation (*70*, *71*). However, it was recently reported that the SUMOylation pathway exhibits cross-talk with ubiquitination via STUbLs to ubiquitinate and tag SUMOylated proteins for proteasomal degradation (*44*, *45*). This SUMO-STUbL–mediated 26*S* proteasomal degradation of target proteins is crucial for DNA damage/genomic integrity (*72*–*74*), transcription/methylation (*75*, *76*), and tumor suppression (*77*, *78*).

On the contrary, our SUMO1/PSME3/20*S* proteasome axis–mediated system is quite in contrast to this system, although both systems use SUMOylation. We provided evidence that the stability of the CP2c TF is negatively regulated by the SUMO1-mediated PSME3/20*S* proteasome pathway in a ubiquitin- and STUbL-independent manner (Fig. 3B and fig. S5). Regarding the proteasomal degradation mechanism by this SUMO1/PSME3/20*S* proteasome axis, it is speculated that SUMO1-mediated SUMOylation may add disorderedness of a target protein suitable for PSME3/20*S* proteasomal degradation because the 11*S* proteasome activator (PSME3)/20*S* proteasome is known to target for degradation of structurally abnormal, misfolded, or highly oxidized proteins (*49*, *50*), and the N- and C-terminal regions of SUMO1 show high “intrinsic disorder” (*26*). In addition, we found that SUMO1-conjugated mono-SUMOylation of CP2c and PSME3 is mainly involved in the PSME3/20*S* proteasomal pathway, although SUMO2/3-conjugated poly-SUMOylation of PSME3 could also be involved in this process (Fig. 4D).

It is well known that SUMO1 acts as a chain terminator, as none of its Lys residues can be further conjugated by any SUMO (*44*) and are mostly used for poly-SUMOylation via internal SUMOylation sites in their flexible N-terminal domains (*30*, *79*), forming homopolymerized or heteropolymerized chains, mainly via conjugation to their conserved N-terminally located residue at K11, which is absent in SUMO1. Therefore, regarding the choice of SUMOylation-dependent degradation via the 26*S* or 20*S* proteasomal pathways, we speculate that it depends on the substrates’ SUMOylation characteristics—while SUMO2/3-mediated poly-SUMOylation is required for the SUMO-STUbL–mediated 26*S* proteasomal degradation, SUMO1-mediated mono-SUMOylation is involved in the SUMO1/PSME3-mediated 20*S* proteasomal pathway.

The major driving force underlying cell cycle progression is the sequential activation of cyclin-dependent kinases (CDKs), which is achieved in part by the ubiquitin-mediated 26*S* proteasomal proteolysis of their cyclin partners and kinase inhibitors. However, SUMOylation also plays critical roles during cell cycle progression, and many important cell cycle regulators, including many oncogenes and tumor suppressors, are functionally regulated via SUMOylation (*45*, *80*). As many of the identified SUMO target proteins are known as oncogenes and tumor suppressors, deregulation of these pathways via overexpression of the SUMO system is known to contribute to increased cell proliferation and cell invasion and reduced apoptosis in tumors.

Our findings are consistent with previous reports that SUMOs have essential functions at each phase of the cell cycle (*45*), and we show that precisely timed degradation of CP2c by SUMO1/PSME3/20*S* proteasome–mediated machinery is essential to ensure accurate progression through the cell cycle. As the *SUMO2* mRNA accounts for most of the entire *SUMO* mRNA pool, SUMO2 would SUMOylate the key cell cycle regulators, including CDKs, topoisomerase II, Nuf2, BubR1, CDCA8, RhoGDIα, and FoxM1, to induce proper cell cycle progression in connection with other protein modification systems (*80*). In addition, the other SUMO2 SUMOylated factors by themselves or partner proteins that interacted with them through the SUMOylation-SIM interaction could also trigger the formation of complexes at centromeric regions, allowing chromosome alignment, condensation, and segregation, and spindle assembly in mitotic stage–specific manner. These SUMOylated proteins would degrade by STUbL system and/or SENP6/7 as completion of mitotic progression. Therefore, the SUMO2 system would function as a major cellular machinery, although the detailed mechanisms underlying these events are not known.

On the contrary, SUMO1 may function to simply turn over nuclear factors during the cell cycle via SUMO1/PSME3/20*S* proteasome–mediated machinery—CP2c should degrade timely and massively after completion of oncogenic transcription function in the S phase, as timely up-regulation of CP2c is required for the G_1_ to S transition. We found that SUMO1/PSME3/20*S* proteasome axis–mediated CP2c degradation is required for proper cell cycle progression. The endogenous and natural CP2c is SUMOylated by SUMO1 during the S to the G_2_-M phase transition, and the SUMOylated CP2c binds to PSME3 in the nuclear envelope to induce CP2c degradation via 20*S* proteasome at the G_2_ phase. As the nuclear membrane breaks down at the M phase, the SUMOylated CP2c could also degrade in the cytoplasm. On the other hand, during the M to the G_0_-G_1_ phase transition, CP2c binds to the cytosolic SUMOylated PSME3, resulting in CP2c degradation in the cytosol. Therefore, the cellular steady-state level of CP2c is expected to be regulated partly by the CP2c transcription rate and CP2c transcriptional complex formation, and also by stage-specific degradation through the SUMO1/PSME3/20*S* proteasome axis. Further efforts to identify the effects of CP2c degradation by cell cycle stage–specific SUMOylation of PSME3 as well as CP2c will provide additional functional insights into the role of CP2c as an anticancer target. It is important to note that SUMO1/PSME3/20*S* proteasome axis–mediated cell stage–specific degradation is not limited to CP2c, as we find additional nuclear factors that are regulated by cell cycle stage–limited degradation through the SUMO1/PSME3/20*S* proteasome axis—these factors are degraded not only by the SUMO1/PSME3/20*S* proteasome axis but also by ubiquitination-dependent proteolysis (Fig. 6 and fig. S11).

There are still many limitations in understanding the SUMO1/PSME3/20*S* proteasome axis–mediated protein degradation. First, further studies are required for elucidation of the detailed molecular mechanisms, such as the distribution of the SUMO machinery proteins and the dynamic signals required for accurate localization of this SUMO machinery during cell cycle progression, and identification of other SUMO1/PSME3/20*S* proteasome targets and elucidation of their cell cycle–dependent regulation mechanism. Second, selectivity in SUMO-SIM interactions of CP2c and PSME3 is not known. Given a large number of SUMO-interacting proteins, it is essential to determine the basis for specificity in SIM-SUMO interactions, an issue that remains largely unaddressed, although several possible explanations have been suggested, including additional binding surfaces in the target protein (*81*), one or more additional protein-protein interactions, and other modulatory posttranslational modification in the protein (*81*, *82*). Third, because a recent paradigm shift in the field of transcriptional regulation has put forward a phase separation model for transcriptional control, in which multimolecular assemblies would form by phase separation bridging enhancers and promoters allowing gene activation (*83*), we need to understand cell cycle stage–specific protein homeostasis through the SUMO1/PSME3/20*S* proteasome pathway in the context of phase separation.

In conclusion, our work provides previously unidentified mechanistic insights into the roles of dynamic degradation of CP2c in cell cycle progression with SUMO1-mediated SUMOylation of CP2c and PSME3, and these SUMOylation events allow CP2c to be spatiotemporally degraded via the 20*S* proteasome. These findings highlight an important function of CP2c signaling in the context of cell cycle progression and open new ways to address other veiled puzzles in the functions of CP2c as a TF and for a better understanding of the functions of the SUMO1-mediated 20*S* proteasomal degradation pathway in general. In addition, we reveal a unique SUMO1-mediated uncanonical 20*S* proteasome degradation mechanism via the SUMO1/PSME3 axis involving mutually alternative SUMOylation of the target protein or PSME3.

## MATERIALS AND METHODS

### Cell culture

The human embryonic kidney 293T (American Type Culture Collection no. CRL-3216) and human breast cancer (the LM1 line of MDA-MB-231, donation from S.-J. Lee) cell lines were maintained in Dulbecco’s modified Eagle’s medium (DMEM; HyClone, SH30243.01) supplemented with 10% fetal bovine serum (FBS; Cellsera, AU-FBS/PG), penicillin (100 U/ml; Sigma-Aldrich, P3032), and streptomycin (100 μg/ml; Sigma-Aldrich, S9137) at 37°C, 5% CO_2_ incubator. For transient transfections, cells were plated at a density of 10^6^ cells/100-mm culture dish 1 day before transfection. Plasmids were transfected into cell lines using the calcium phosphate method or Effectene reagent (Qiagen, 301425), and small interfering RNAs (siRNAs) (listed in table S1) were transfected into cell lines using Lipofectamine (Thermo Fisher Scientific, 11668027). For doxycycline-inducible gene expression regulation, cell lines were incubated in the presence of doxycycline (1 μg/ml; Sigma-Aldrich, D1822) for 2 to 3 days. For the protein degradation inhibition experiments, cells were treated with the proteasomal inhibitor MG132 (Sigma-Aldrich, 474790) or the lysosomal inhibitor E64 (Sigma-Aldrich, 324890). For the protein synthesis inhibition experiments, cells were treated with CHX (Sigma-Aldrich, 239764). For the SUMOylation and/or ubiquitination inhibition experiments, cells were treated with the SUMO E1 inhibitor TAK981 (Selleckchem, S8829) and/or the ubiquitin E1 inhibitor TAK243 (Selleckchem, S8341). For activation of STUbL (RNF111)–mediated protein degradation, cells were treated with transforming growth factor–β (TGF-β) (1 ng/ml) (Invitrogen, 10355488) for 6 hours. For the single thymidine block, cells were incubated in a medium containing thymidine (2 mM) for 19 hours. For the double thymidine block (synchronization at G_1_-S phase), cells were incubated in a medium containing thymidine (2 mM) for 19 hours, released in a fresh medium for 10 hours, and incubated in a thymidine medium (2 mM) for 19 hours. To synchronize with the G_2_-M phase, cells were incubated in a medium containing thymidine (2 mM) for 19 hours and incubated in a medium containing nocodazole (100 ng/ml) for 16 hours.

### Plasmid construction

For the yeast two-hybrid experiment, the Eco RI/Sal I–digested 306-502 fragment of CP2c from pT7-Blue-CP2c was subcloned into the pLexA yeast vector. For the construction of glutathione *S*-transferase (GST)–tagged CP2c bacterial expression vector, the CP2c complementary DNA (cDNA) was amplified from mRNA of the mouse erythroleukemia cells by various combinations of polymerase chain reaction (PCR) primers. The amplified cDNA was digested with Eco RI/Sal I and subcloned into the pGEX-2TK vector. For the construction of the GST-tagged SUMO1 bacterial expression vector, the Bam HI/Xho I-digested fragment of SUMO1 from pCMV-HA-SUMO1 was subcloned into the Bam HI/Sal I site in the pGEX-4T1 vector. The pCMV-HA-CP2c expression vector was generated by cloning the Sal I/Kpn I–digested fragment of CP2c from pT7-Blue-CP2c into the pCMV-HA vector. The Flag-tagged serial deletion mutant CP2c was amplified by various combinations of PCR primers. The amplified cDNA was cloned into the Eco RI/Xho I site of the pcDNA3-Flag vector. The Flag-tagged TEL was generated by cloning the Eco RI-digested fragment of TEL from pcDNA3-His-TEL into the pcDNA3-Flag vector.

For the construction of pcDNA3-Flag-SUMO1, the partially digested Xho I/Eco RI fragment from the pB42AD-SUMO1 vector was inserted into the Flag-tagged pcDNA3. The pEGFP-SUMO1 (ΔGG) vector containing C-terminal deletion of two glycine residues at G_96_ and G_97_ was generated by inserting PCR-amplified SUMO1 open reading frame from pEGFP-SUMO1 into the pEGFP-C1 vector. A PCR-based site-directed mutagenesis approach was used to generate point mutants in the SIM domains or SUMOylation sites of CP2c and PSME3 and in the SIM interaction region (i.e., H35A/F36A/V38A) of SUMO1. Oligonucleotides containing the shRNA sequence with Bgl II and Hind III restriction enzyme sites that were compatible with cloning into the pSuper puro vector were designed. Annealed double-strand oligonucleotides in annealing buffer [100 mM NaCl and 50 mM Hepes (pH 7.4)] were ligated into the predigested pSuper puro plasmid. For doxycycline-inducible expression of shRNA, H1 promoter and shRNA were subcloned into pDUAL-tet I/O plasmid using Eco RI and Cla I.

For the in vitro FRET experiments, PCR products of CP2c WT, SIM 158m, or K50R, and PSME3 WT, 6KR, and double SIM mutant (SIM 70 and SIM115) were cloned into the pRSET-eYFP-cas-dTomato vector using Bam HI and Eco RI or Kpn I and Hind III, respectively. For the in vivo FRET experiments, PCR products of CP2c WT and PSME3 WT were cloned into the eYFP-N1 vector using Bgl II and Eco RI or the mCherry-PSME3-pcDNA3 vector using Xho I and Spe I, respectively. Primer sequences for PCR and the specific synthetic oligonucleotides used were listed in table S1.

We thank the following people for providing valuable constructs or research materials: S. Y. Choe (for pcDNA3-His-hTEL), J. S. Lee (for pGEX-SUMO1, pGEX-UBE2I, pcDNA3-Flag-UBE2I, pCMV-HA-SUMO1, and pEGFP-SUMO1), J. J. Palvimo [for pEGFP-SUMO(G97A)], S. Hong (for pcDNA3-His-ubiquitin), C.-Y. Choi (for EGFP-SUMO2 and EGFP-SUMO3), and G. Suske (for pGEX-2TK-PIAS1).

### Yeast two-hybrid screening

Yeast two-hybrid screening was performed using the MATCHMAKER System (Takara, 630489). The interaction was analyzed for β-galactosidase activity by filter lift experiments (*84*) and then quantified by *o*-nitrophenyl-d-galactopyranoside assays as described (*85*).

### Purification of recombinant bacterial proteins and GST pull-down assay

For expression of GST tag or His tag fusion proteins, BL21 (DE3) pLysS strain cells were grown in Luria Bertani supplemented with ampicillin (100 μg/ml). Cells were grown at 37°C while shaken at 200 rpm until the OD_600_ (optical density at 600 nm) value reached between 0.4 and 0.6. Expression was induced with 0.4 mM isopropyl-β-d-thiogalactopyranoside (IPTG), and expression conditions were optimized for individual constructs. Cells were lysed in lysis buffer [50 mM Tris-HCl (pH7.4), 150 mM NaCl, 1 mM EDTA, and 1 mM phenylmethylsulfonyl fluoride (PMSF)] for 30 min, sonicated two times with four pulses, placed on ice for 10 s, and purified by glutathione–Sepharose 4B beads (Sigma-Aldrich, GE17-0756-01) or Ni–nitrilotriacetic acid agarose beads (Qiagen, 30210). For the in vitro GST pull-down, 293T cell lysates were incubated for 3 hours at 4°C with GST and bound to the glutathione–Sepharose beads. After washing three times with lysis buffer, proteins were eluted by boiling in 2× SDS–polyacrylamide gel electrophoresis (SDS-PAGE) loading buffer and subjected to SDS-PAGE. For the His-tagging protein purification, beads were washed with 1 ml of washing buffer (50 mM NaH_2_PO_4_, 300 mM NaCl, 20 mM imidazole, and 1 mM PMSF), and His-fusion proteins were eluted with elution buffer (50 mM NaH_2_PO_4_, 300 mM NaCl, 250 mM imidazole, and 1 mM PMSF).

### Co-IP assay

Cell lysates were harvested in lysis buffer [50 mM Tris-HCl (pH7.4), 150 mM NaCl, 1 mM EDTA, 1% NP-40, and 1 mM PMSF] with freshly added 1 mM dithiothreitol (DTT) and protease inhibitor cocktail (Sigma-Aldrich, P8340). For protein degradation assays, various expression plasmids with different tags and mutations were transfected singly or in combinations into 293T cells and treated with MG132 (50 μM) for 12 hours before being harvested. Input samples (10% of lysate) were saved for Western blot analysis. For normal IP, precleared extracts were incubated with 2 μg of antibodies (listed in table S2) and protein A/G agarose beads (Santa Cruz Biotechnology, sc-2003) by rotating overnight at 4°C. The immune complexes were washed three times with lysis buffer; bound proteins were eluted with 2× bed volume of 0.2 M glycine buffer, followed by neutralization with an equal volume of 1 M Tris-HCl (pH 8.0). For Flag-tag IP, precleared extracts were incubated with Flag-M2 bead (Sigma-Aldrich, A2220) by rotating overnight at 4°C. The immune complexes were washed three times with lysis buffer, and bound proteins were eluted with Flag peptide (100 μg/ml; Sigma-Aldrich, F4799). Precipitated proteins were analyzed using Western blot.

### Western blot

Total cellular extracts were prepared using the lysis buffer [50 mM tris-HCl (pH 7.4), 150 mM NaCl, 1 mM EDTA, 0.1% Triton X-100, and 1 mM PMSF]. For protein degradation assays, various expression plasmids with different tags and mutations were transfected singly or in combinations into 293T cells and treated with MG132 (10 or 50 μM) at various times before being harvested. To accurately determine the SUMOylation pattern of CP2c or PSME3, the lysate was extracted by adding 25 mM NEM to the lysis buffer. The protein lysates were separated by SDS-PAGE and electroblotted onto polyvinylidene difluoride membranes (GE Healthcare, 10600069). After blocking with PBS-T (phosphate-buffered saline–0.1% Tween 20) containing 5% nonfat dry milk, membranes were incubated with the indicated primary antibodies (listed in table S2) overnight at 4°C. Horseradish peroxidase–conjugated secondary antibodies were treated for 1 hour at room temperature. The polyclonal anti-ACTB or anti–β-tubulin antibody was used as the loading control for the Western blot. Protein expression was visualized by chemiluminescence using an ECL system (GE Healthcare, RPN2106). Relative amounts of proteins were quantified using ImageJ (version 1.51) software.

### Metabolic labeling and IP

Cell labeling was performed after ~1 hour of preincubation in methionine- and cysteine-free RPMI containing dialyzed 10% FBS. Cells were incubated with [^35^S]cysteine/methionine (200 μCi/ml; 7900 Ci/mmol; Amersham Pharmacia Biotech, Little Chalfont, UK) for 1 hour, washed three times in PBS, and chased with complete RPMI for different periods of time. After IP with an anti-CP2c antibody, proteins were fractionated by 10% SDS-PAGE and the dried gels were autoradiographed.

### Reverse transcription PCR

Total cellular RNA was isolated using QIAzol reagent (Qiagen, 79306) according to the manufacturer’s procedures. Purified RNA was dissolved in diethylpyrocarbonate water. Reverse transcription (RT) was performed using a High-Capacity cDNA Reverse Transcription kit (Toyobo, FSQ-201) in the presence of 400 ng of total RNA and 10 pmol of the random hexamer. PCR was performed using a rTaq Plus 5× PCR master mix (Elpis-Biotech, EBT-1319). Oligonucleotide primers used in RT-PCR are listed in table S1.

### Dual luciferase assay

A luciferase reporter construct containing tetrameric CP2c half-binding sites (CP2c-tet) was used for luciferase assays. 293T cells were transiently transfected with 400 ng of DNA, including both the luciferase reporter construct and various combinations of CP2c, CP2b, SUMO1, UBE2I, PIAS1, and PSME3 expression vectors using calcium phosphate or Effectene method. The transfection ratio of the CP2c-tet-firefly luciferase vector and the renilla luciferase vector was 5:1. Cells were harvested 48 hours after transfection with 100 μl of passive lysis buffer (Promega, E1941). To estimate luciferase activity, 20-μl aliquots of each lysate were used to quantify using the dual luciferase reporter assay system (Promega, E1910) on the Lumat LB9501 Luminometer (Berthold). Firefly luciferase activity was normalized against renilla luciferase, and the data were represented as the ratio of firefly to renilla luciferase activity (Fluc/Rluc).

### Fluorescent and immunofluorescent microscopy

For immunofluorescence, 293T cells were grown as a monolayer for 2 days on glass coverslips in a 24-well plate. Cell cycle–based sorted 293T cells were attached to the poly-l-lysine–coated slide glasses in Cytospin 4 (Thermo Fisher Scientific). Cells were fixed in coverslips or slide glasses with 4% paraformaldehyde for 15 min, permeabilized with 0.1% Triton X-100 for 10 min, rinsed with PBS, and blocked in PBS containing 0.05% Triton X-100 and 3% horse serum (Gibco, 16050-122) for 1 hour at room temperature. Fixed cells were incubated with the indicated primary monoclonal antibodies (listed in table S2) in PBS containing 1% bovine serum albumin (BSA) solution overnight at 4°C. To the cells rinsed with PBS, the corresponding fluorescein isothiocyanate (FITC)– or Cy3-conjugated secondary antibodies (listed in table S2) were added in PBS containing 1% BSA solution for 1 hour at room temperature. Antibody-labeled cells were mounted with a mounting solution containing 4′,6-diamidino-2-phenylindole (DAPI) (Vector Laboratories, H1200-10). Images obtained with a fluorescent microscope or a confocal laser-scanning microscope (Nikon) were analyzed using ImageJ software (version 1.51).

### FRET assay

For the in vitro FRET assay, the donor (eYFP-CP2c WT, SIM 158m, and K50R) and receptor (PSME3 WT, 6KR, and double SIM mutant-dTomato) protein-containing lysate were extracted from the IPTG-induced BL21(DE3) pLysS strain. EYFP-CP2c WT and PSME3 WT-dTomato proteins were conjugated with SUMO1, SUMO2, or SUMO3 using an in vitro SUMOylation kit (Abcam, 139470) according to the manufacturer’s procedures. EYFP-CP2c WT with or without SUMOylation was mixed with each receptor, whereas PSME3 WT-dTomato with or without SUMOylation was mixed with each donor. Protein mixtures were incubated at 37°C for 30 min and seeded on a 96-well plate. Emission spectra from 500 to 600 nm, upon excitation at 480 nm with a bandwidth of 2 nm, were obtained in the Varioskan Flash Spectral Scanning Multimode Reader (Thermo Fisher Scientific). The intensities of each experimental sample were normalized to average emission for dTomato stimulation in samples where fluorescent proteins were not included. The mean FRET efficiency (*R*_FRET_) was calculated as *R*_FRET_ = IA/(IA + ID), where IA and ID represent acceptor and donor intensities, respectively. The reactants used in the in vitro FRET assay were reverified by co-IP Western blot.

For the FRET assay in the cells, 293T cells were grown on glass-bottom dishes (SPL Life Sciences, 200350) in DMEM supplemented with 10% FBS (HyClone, SH30084.03) and then transfected with plasmids (dTomato-PSME3 and eYFP-CP2c) using Effectene (Qiagen, 301425). From 12 hours after transfection, the cell cycle was arrested at G_1_-S or G_2_-M, and MG132 was treated with 50 μM 6 hours before the cell cycle was released. FRET imaging was performed by confocal microscopy (Nikon C2si) after replacing the culture medium with DMEM without phenol red (Welgene, LM001-10). For the in-cell FRET analysis, the donor fluorescence was excited at 488 nm by a laser, and the emission of the acceptor was collected through a 570/613-nm filter (Nikon, 67-006-NKN). Excitation and emission for eYFP fluorescence were 488 and 525/561 nm, whereas those were 550 and 570/613 nm for dTomato fluorescence, respectively. For the quantification of FRET efficiency, an emission value at 588 nm, upon excitation at 488 nm, was obtained in the Varioskan Flash Spectral Scanning Multimode Reader (Thermo Fisher Scientific). Image processing was performed using the ImageJ software (version 1.51). The relative FRET ratios for compounds were calculated by FRETcomp/FRETmock.

### In vitro protein degradation assay in a reconstituted cell-free system

In vitro SUMOylation and in vitro ubiquitination of bead-bound GST-CP2c were carried out with a SUMOylation kit (Enzo Life Sciences, BML-UW8955-0001) or Ubiquitination kit (Enzo Life Sciences, BML-UW9920-0001), respectively, according to the manufacturer’s procedures. Following 1-hour incubation at 37°C, the reaction was terminated by GST pull down. The modified GST-CP2c samples were incubated with 20*S* proteasome (Enzo Life Sciences, BML-AK740-0001) or 26*S* proteasome (Enzo Life Sciences, BML-PW8950-0001) in each assay buffer. After 1 hour of incubation at 37°C, the reaction was terminated with an SDS loading buffer. The samples prepared above were analyzed using Western blot.

### DSP crosslinking

For determination of CP2c-SUMO1-PSME3–containing complexes, live cells were washed twice with the 2.5 mM sodium phosphate (pH 7.4) buffer and crosslinked using 2 mM DSP in PBS for 3 hours on the ice, and crosslinking was terminated with 50 mM Tris-HCl (pH 7.4) for 15 min at room temperature. After intracellular DSP crosslinking, cells were lysed in non–tris-based lysis buffer (50 mM HEPES, 150 mM NaCl, 1 mM EDTA, 1% NP-40, and 1 mM PMSF) for 20 min at 4°C. Various samples were incubated overnight at 4°C with the appropriate antibodies for co-IP. Half of each sample (pull-down sample and immunoprecipitated sample) was analyzed together with the crosslinker cleaved by adding DTT (final 50 mM) by Western blot or MS.

### Cell cycle analysis

Cells were washed with prechilled PBS, fixed with prechilled 70% ethanol for 30 min at ice-cold temperature, and stored overnight at −20°C freezer. Before analysis, fixed cells were incubated with ribonuclease A (20 μg/ml) and stained with propidium iodide staining solution at room temperature for 20 min under dark conditions. Samples were immediately analyzed on BD FACSCanto II using BD FACSDiva software (BD Biosciences). The cell fractions in sub-G_1_, G_0_-G_1_, S, G_2_/M, and polyploidy states were quantified in histograms with FlowJo software (BD Biosciences).

### MS analysis

To analyze the binding pattern between proteins by matrix-assisted laser desorption/ionization–time-of-flight (MALDI-TOF), DSP crosslinking, and IP or pull-down targeting the protein of interest performed on the protein of interest as described above. Each sample was alkylated by incubating with iodoacetamide (10 μg/μl final) for 20 min in the dark and precipitated using 10% trichloroacetic acid. Sequencing-grade modified trypsin (0.3 μg) (Promega, V5111) was added to each sample and allowed to incubate overnight at 37°C. Each sample was analyzed on a Shimadzu Axima MALDI-TOF mass spectrometer. One microliter of each digested sample in 30% acetonitrile and 50 mM NH_4_HCO_3_ was spotted on a MALDI plate in a sandwich-style manner with α-cyano-4-hydroxycinnamic acid matrix (10 mg/ml) at a total ratio of matrix and sample (1:1). All MS spectra were acquired in linear mode with an average of 300 profiles per sample and an average power of 90. All peptide molecular weights were identified using DataExplorer (Applied Biosystems) and analyzed using ExPasy (PeptideMass and MASCOT).

All branch peptides were identified manually. To analyze the SUMOylation pattern of proteins, the lysine of protein of interest capable of SUMOylation was predicted using GPS-SUMO1 1.0 software, and the mass of each peptide fragment derived from lysine conjugated to was calculated. We analyzed whether the mass peak specifically observed in the SUMOylation-induced sample matches the predicted mass of the SUMO1-conjugated lysine peptide fragment.

To analyze the binding patterns between proteins (SIM domain and protein-protein interaction), MALDI-TOF analysis was performed using a DSP crosslinking sample and a decrosslinking sample treated with DTT after crosslinking. The analysis was carried out in the following steps: (i) observe whether the abnormally large mass peak caused by DSP crosslinking disappears after DTT treatment in the region where proteins are closely bound to each other and (ii) observe and analyze the combination of mass peaks specifically observed only after DTT treatment and whether they correspond to the previously observed abnormal peaks.

Predicted mass/charge (*m*/*z*) ratios were searched in the raw data, and fragment ions of those precursor *m*/*z* ratios in corresponding MS/MS spectra were matched to the theoretical fragments.

### Tertiary structure prediction

Tertiary structures of CP2c, SUMO1 and PSME3 were obtained from the AlphaFold protein structure database (*86*). HADDOCK was used to generate the structural models for CP2c-SUMO1-PSME3–containing complexes in combination with DSP crosslinking MS (*87*). All structures were visualized using PyMOL software.

### Statistical analysis

Data are presented as means ± SE. The sample size for each experiment, *n*, was included in the Results and the associated figure legend. Everywhere in the text, the difference between two subsets of data was considered statistically significant if the one-tailed Student’s *t*-test gave a significance level *P* (*P* value) less than 0.05. Statistical analysis was performed in GraphPad Prism 6.

## Notes

View/request a protocol for this paper from *Bio-protocol*.

## References

[1] H. C. Kang, J. H. Chae, Y. H. Lee, M.-A. Park, J. H. Shin, S.-H. Kim, S.-K. Ye, Y. S. Cho, S. Fiering, C. G. Kim, Erythroid cell-specific α-globin gene regulation by the CP2 transcription factor family. Mol. Cell. Biol. 25, 6005–6020 (2005). 15988015 10.1128/MCB.25.14.6005-6020.2005 PMC1168829

[2] S. Rodda, S. Sharma, M. Scherer, G. Chapman, P. Rathjen, CRTR-1, a developmentally regulated transcriptional repressor related to the CP2 family of transcription factors. J. Biol. Chem. 276, 3324–3332 (2001). 11073954 10.1074/jbc.M008167200

[3] T. Wilanowski, A. Tuckfield, L. Cerruti, S. O’Connell, R. Saint, V. Parekh, J. Tao, J. M. Cunningham, S. M. Jane, A highly conserved novel family of mammalian developmental transcription factors related to *Drosophila grainyhead*. Mech. Dev. 114, 37–50 (2002). 12175488 10.1016/s0925-4773(02)00046-1

[4] J. B. Yoon, G. Li, R. G. Roeder, Characterization of a family of related cellular transcription factors which can modulate human immunodeficiency virus type 1 transcription in vitro. Mol. Cell. Biol. 14, 1776–1785 (1994). 8114710 10.1128/mcb.14.3.1776-1785.1994 PMC358535

[5] S. M. Jane, A. W. Nienhuis, J. M. Cunningham, Hemoglobin switching in man and chicken is mediated by a heteromeric complex between the ubiquitous transcription factor CP2 and a developmentally specific protein. EMBO J. 14, 97–105 (1995). 7828600 10.1002/j.1460-2075.1995.tb06979.x PMC398056

[6] C. G. Kim, S. L. Swendeman, K. M. Barnhart, M. Sheffery, Promoter elements and erythroid cell nuclear factors that regulate α-globin gene transcription in vitro. Mol. Cell. Biol. 10, 5958–5966 (1990). 2233727 10.1128/mcb.10.11.5958-5966.1990 PMC361393

[7] G. Kotarba, E. Krzywinska, A. I. Grabowska, A. Taracha, T. Wilanowski, TFCP2/TFCP2L1/UBP1 transcription factors in cancer. Cancer Lett. 420, 72–79 (2018). 29410248 10.1016/j.canlet.2018.01.078

[8] Y.-P. Cheon, D. Choi, S.-H. Lee, C. G. Kim, YY1 and CP2c in unidirectional spermatogenesis and stemness. Dev. Reprod. 24, 249–262 (2020). 33537512 10.12717/DR.2020.24.4.249 PMC7837418

[9] J. Veljkovic, U. Hansen, Lineage-specific and ubiquitous biological roles of the mammalian transcription factor LSF. Gene 343, 23–40 (2004). 15563829 10.1016/j.gene.2004.08.010 PMC3402097

[10] J. H. Chae, C. G. Kim, CP2 binding to the promoter is essential for the enhanced transcription of globin genes in erythroid cells. Mol. Cells 15, 40–47 (2003). 12661759

[11] P. Bruni, G. Minopoli, T. Brancaccio, M. Napolitano, R. Faraonio, N. Zambrano, U. Hansen, T. Russo, Fe65, a ligand of the Alzheimer’s β-amyloid precursor protein, blocks cell cycle progression by down-regulating thymidylate synthase expression. J. Biol. Chem. 277, 35481–35488 (2002). 12089154 10.1074/jbc.M205227200

[12] C. M. Powell, T. L. Rudge, Q. Zhu, L. F. Johnson, U. Hansen, Inhibition of the mammalian transcription factor LSF induces S-phase-dependent apoptosis by downregulating thymidylate synthase expression. EMBO J. 19, 4665–4675 (2000). 10970859 10.1093/emboj/19.17.4665 PMC302058

[13] C. Solis, G. I. Aizencang, K. H. Astrin, D. F. Bishop, R. J. Desnick, Uroporphyrinogen III synthase erythroid promoter mutations in adjacent GATA1 and CP2 elements cause congenital erythropoietic porphyria. J. Clin. Invest. 107, 753–762 (2001). 11254675 10.1172/JCI10642 PMC208941

[14] P. K. Santhekadur, D. Rajasekaran, A. Siddiq, R. Gredler, D. Chen, S. E. Schaus, U. Hansen, P. B. Fisher, D. Sarkar, The transcription factor LSF: A novel oncogene for hepatocellular carcinoma. Am. J. Cancer Res. 2, 269–285 (2012). 22679558 PMC3365805

[15] F. Romerio, M. N. Gabriel, D. M. Margolis, Repression of human immunodeficiency virus type 1 through the novel cooperation of human factors YY1 and LSF. J. Virol. 71, 9375–9382 (1997). 9371597 10.1128/jvi.71.12.9375-9382.1997 PMC230241

[16] Y. Zhao, N. Kaushik, J.-H. Kang, N. K. Kaushik, S. H. Son, N. Uddin, M.-J. Kim, C. G. Kim, S.-J. Lee, A feedback loop comprising EGF/TGFα sustains TFCP2-mediated breast cancer progression. Cancer Res. 80, 2217–2229 (2020). 32193292 10.1158/0008-5472.CAN-19-2908

[17] B. K. Yoo, L. Emdad, R. Gredler, C. Fuller, C. I. Dumur, K. H. Jones, C. Jackson-Cook, Z.-Z. Su, D. Chen, U. H. Saxena, U. Hansen, P. B. Fisher, D. Sarkar, Transcription factor late SV40 factor (LSF) functions as an oncogene in hepatocellular carcinoma. Proc. Natl. Acad. Sci. U.S.A. 107, 8357–8362 (2010). 20404171 10.1073/pnas.1000374107 PMC2889542

[18] S. H. Son, M. Y. Kim, E. Jo, V. N. Uversky, C. G. Kim, Structural and functional insights into CP2c transcription factor complexes. Int. J. Mol. Sci. 23, 6369 (2022). 35742810 10.3390/ijms23126369 PMC9223585

[19] H. C. Kang, J. H. Chae, J. Jeon, W. Kim, D. H. Ha, J. H. Shin, C. G. Kim, C. G. Kim, PIAS1 regulates CP2c localization and active promoter complex formation in erythroid cell-specific α-globin expression. Nucleic Acids Res. 38, 5456–5471 (2010). 20421208 10.1093/nar/gkq286 PMC2938217

[20] H. C. Kang, B. M. Chung, J. H. Chae, S.-I. Yang, C. G. Kim, C. G. Kim, Identification and characterization of four novel peptide motifs that recognize distinct regions of the transcription factor CP2. FEBS J. 272, 1265–1277 (2005). 15720400 10.1111/j.1742-4658.2005.04564.x

[21] J. R. Gareau, C. D. Lima, The SUMO pathway: Emerging mechanisms that shape specificity, conjugation and recognition. Nat. Rev. Mol. Cell Biol. 11, 861–871 (2010). 21102611 10.1038/nrm3011 PMC3079294

[22] J. Zhao, Sumoylation regulates diverse biological processes. Cell. Mol. Life Sci. 64, 3017–3033 (2007). 17763827 10.1007/s00018-007-7137-4 PMC7079795

[23] D. Baczyk, M. C. Audette, S. Drewlo, K. Levytska, J. C. Kingdom, SUMO-4: A novel functional candidate in the human placental protein SUMOylation machinery. PLOS ONE 12, e0178056 (2017). 28545138 10.1371/journal.pone.0178056 PMC5435238

[24] S. Chen, T. Yang, F. Liu, H. Li, Y. Guo, H. Yang, J. Xu, J. Song, Z. Zhu, D. Liu, Inflammatory factor-specific sumoylation regulates NF-κB signalling in glomerular cells from diabetic rats. Inflamm. Res. 63, 23–31 (2014). 24173240 10.1007/s00011-013-0675-3

[25] C. Y. Wang, J.-X. She, SUMO4 and its role in type 1 diabetes pathogenesis. Diabetes Metab. Res. Rev. 24, 93–102 (2008). 17990297 10.1002/dmrr.797

[26] A. B. Celen, U. Sahin, Sumoylation on its 25th anniversary: Mechanisms, pathology, and emerging concepts. FEBS J. 287, 3110–3140 (2020). 32255256 10.1111/febs.15319

[27] V. Bernier-Villamor, D. A. Sampson, M. J. Matunis, C. D. Lima, Structural basis for E2-mediated SUMO conjugation revealed by a complex between ubiquitin-conjugating enzyme Ubc9 and RanGAP1. Cell 108, 345–356 (2002). 11853669 10.1016/s0092-8674(02)00630-x

[28] G. Gill, SUMO and ubiquitin in the nucleus: Different functions, similar mechanisms? Genes Dev. 18, 2046–2059 (2004). 15342487 10.1101/gad.1214604

[29] G. W. Lee, F. Melchior, M. J. Matunis, R. Mahajan, Q. Tian, P. Anderson, Modification of Ran GTPase-activating protein by the small ubiquitin-related modifier SUMO-1 requires Ubc9, an E2-type ubiquitin-conjugating enzyme homologue. J. Biol. Chem. 273, 6503–6507 (1998). 9497385 10.1074/jbc.273.11.6503

[30] M. H. Tatham, E. Jaffray, O. A. Vaughan, J. M. P. Desterro, C. H. Botting, J. H. Naismith, R. T. Hay, Polymeric chains of SUMO-2 and SUMO-3 are conjugated to protein substrates by SAE1/SAE2 and Ubc9. J. Biol. Chem. 276, 35368–35374 (2001). 11451954 10.1074/jbc.M104214200

[31] I. Matic, B. Macek, M. Hilger, T. C. Walther, M. Mann, Phosphorylation of SUMO-1 occurs in vivo and is conserved through evolution. J. Proteome Res. 7, 4050–4057 (2008). 18707152 10.1021/pr800368m

[32] H. Saitoh, J. Hinchey, Functional heterogeneity of small ubiquitin-related protein modifiers SUMO-1 versus SUMO-2/3. J. Biol. Chem. 275, 6252–6258 (2000). 10692421 10.1074/jbc.275.9.6252

[33] A. C. Vertegaal, J. S. Andersen, S. C. Ogg, R. T. Hay, M. Mann, A. I. Lamond, Distinct and overlapping sets of SUMO-1 and SUMO-2 target proteins revealed by quantitative proteomics. Mol. Cell. Proteomics 5, 2298–2310 (2006). 17000644 10.1074/mcp.M600212-MCP200

[34] D. Mukhopadhyay, M. Dasso, Modification in reverse: The SUMO proteases. Trends Biochem. Sci. 32, 286–295 (2007). 17499995 10.1016/j.tibs.2007.05.002

[35] J. S. Seeler, A. Dejean, Nuclear and unclear functions of SUMO. Nat. Rev. Mol. Cell Biol. 4, 690–699 (2003). 14506472 10.1038/nrm1200

[36] A. Verger, J. Perdomo, M. Crossley, Modification with SUMO. EMBO Rep. 4, 137–142 (2003). 12612601 10.1038/sj.embor.embor738 PMC1315836

[37] F. Galli, M. Rossi, Y. D’Alessandra, M. de Simone, T. Lopardo, Y. Haupt, O. Alsheich-Bartok, S. Anzi, E. Shaulian, V. Calabrò, G. La Mantia, L. Guerrini, MDM2 and Fbw7 cooperate to induce p63 protein degradation following DNA damage and cell differentiation. J. Cell Sci. 123, 2423–2433 (2010). 20571051 10.1242/jcs.061010

[38] D.-Y. Lin, Y.-S. Huang, J.-C. Jeng, H.-Y. Kuo, C.-C. Chang, T.-T. Chao, C.-C. Ho, Y.-C. Chen, T.-P. Lin, H.-I. Fang, C.-C. Hung, C.-S. Suen, M.-J. Hwang, K.-S. Chang, G. G. Maul, H.-M. Shih, Role of SUMO-interacting motif in Daxx SUMO modification, subnuclear localization, and repression of sumoylated transcription factors. Mol. Cell 24, 341–354 (2006). 17081986 10.1016/j.molcel.2006.10.019

[39] E. Meulmeester, F. Melchior, SUMO. Nature 452, 709–711 (2008). 18401402 10.1038/452709a

[40] J. Zhu, S. Zhu, C. M. Guzzo, N. A. Ellis, K. S. Sung, C. Y. Choi, M. J. Matunis, Small ubiquitin-related modifier (SUMO) binding determines substrate recognition and paralog-selective SUMO modification. J. Biol. Chem. 283, 29405–29415 (2008). 18708356 10.1074/jbc.M803632200 PMC2570875

[41] R. Vyas, R. Kumar, F. Clermont, A. Helfricht, P. Kalev, P. Sotiropoulou, I. A. Hendriks, E. Radaelli, T. Hochepied, C. Blanpain, A. Sablina, H. van Attikum, J. V. Olsen, A. G. Jochemsen, A. C. O. Vertegaal, J.-C. Marine, RNF4 is required for DNA double-strand break repair in vivo. Cell Death Differ. 20, 490–502 (2013). 23197296 10.1038/cdd.2012.145 PMC3569989

[42] J. Zhao, B. Zhai, S. P. Gygi, A. L. Goldberg, mTOR inhibition activates overall protein degradation by the ubiquitin proteasome system as well as by autophagy. Proc. Natl. Acad. Sci. U.S.A. 112, 15790–15797 (2015). 26669439 10.1073/pnas.1521919112 PMC4703015

[43] M.-C. Geoffroy, R. T. Hay, An additional role for SUMO in ubiquitin-mediated proteolysis. Nat. Rev. Mol. Cell Biol. 10, 564–568 (2009). 19474794 10.1038/nrm2707

[44] A. M. Sriramachandran, R. J. Dohmen, SUMO-targeted ubiquitin ligases. Biochim. Biophys. Acta 1843, 75–85 (2014). 24018209 10.1016/j.bbamcr.2013.08.022

[45] A. C. O. Vertegaal, Signalling mechanisms and cellular functions of SUMO. Nat. Rev. Mol. Cell Biol. 23, 715–731 (2022). 35750927 10.1038/s41580-022-00500-y

[46] A. Plechanovová, E. G. Jaffray, M. H. Tatham, J. H. Naismith, R. T. Hay, Structure of a RING E3 ligase and ubiquitin-loaded E2 primed for catalysis. Nature 489, 115–120 (2012). 22842904 10.1038/nature11376 PMC3442243

[47] A. M. Sriramachandran, K. Meyer-Teschendorf, S. Pabst, H. D. Ulrich, N. H. Gehring, K. Hofmann, G. J. K. Praefcke, R. J. Dohmen, Arkadia/RNF111 is a SUMO-targeted ubiquitin ligase with preference for substrates marked with SUMO1-capped SUMO2/3 chain. Nat. Commun. 10, 3678 (2019). 31417085 10.1038/s41467-019-11549-3 PMC6695498

[48] V. Laigle, F. Dingli, S. Amhaz, T. Perron, M. Chouchène, S. Colasse, I. Petit, P. Poullet, D. Loew, C. Prunier, L. Levy, Quantitative ubiquitylome analysis reveals the specificity of RNF111/Arkadia E3 ubiquitin ligase for its degradative substrates SKI and SKIL/SnoN in TGF-β signaling pathway. Mol. Cell. Proteomics 20, 100173 (2021). 34740826 10.1016/j.mcpro.2021.100173 PMC8665411

[49] G. Ben-Nissan, M. Sharon, Regulating the 20*S* proteasome ubiquitin-independent degradation pathway. Biomolecules 4, 862–884 (2014). 25250704 10.3390/biom4030862 PMC4192676

[50] F. Kumar Deshmukh, D. Yaffe, M. A. Olshina, G. Ben-Nissan, M. Sharon, The contribution of the 20S proteasome to proteostasis. Biomolecules 9, 190 (2019). 31100951 10.3390/biom9050190 PMC6571867

[51] B. M. Stadtmueller, C. P. Hill, Proteasome activators. Mol. Cell 41, 8–19 (2011). 21211719 10.1016/j.molcel.2010.12.020 PMC3040445

[52] W. Dubiel, G. Pratt, K. Ferrell, M. Rechsteiner, Purification of an 11*S* regulator of the multicatalytic protease. J. Biol. Chem. 267, 22369–22377 (1992). 1429590

[53] R. Shringarpure, T. Grune, J. Mehlhase, K. J. A. Davies, Ubiquitin conjugation is not required for the degradation of oxidized proteins by proteasome. J. Biol. Chem. 278, 311–318 (2003). 12401807 10.1074/jbc.M206279200

[54] Y. Wu, L. Wang, P. Zhou, G. Wang, Y. Zeng, Y. Wang, J. Liu, B. Zhang, S. Liu, H. Luo, X. Li, Regulation of REGγ cellular distribution and function by SUMO modification. Cell Res. 21, 807–816 (2011). 21445096 10.1038/cr.2011.57 PMC3085583

[55] H. Sun, J. D. Leverson, T. Hunter, Conserved function of RNF4 family proteins in eukaryotes: Targeting a ubiquitin ligase to SUMOylated proteins. EMBO J. 26, 4102–4112 (2007). 17762864 10.1038/sj.emboj.7601839 PMC2230674

[56] X. Li, L. Amazit, W. Long, D. M. Lonard, J. J. Monaco, B. W. O’Malley, Ubiquitin- and ATP-independent proteolytic turnover of p21 by the REGγ-proteasome pathway. Mol. Cell 26, 831–842 (2007). 17588518 10.1016/j.molcel.2007.05.028

[57] X. Chen, L. F. Barton, Y. Chi, B. E. Clurman, J. M. Roberts, Ubiquitin-independent degradation of cell-cycle inhibitors by the REGγ proteasome. Mol. Cell 26, 843–852 (2007). 17588519 10.1016/j.molcel.2007.05.022 PMC2031223

[58] C. Realini, C. C. Jensen, Z. G. Zhang, S. C. Johnston, J. R. Knowlton, C. P. Hill, M. Rechsteiner, Characterization of recombinant REGα, REGβ, and REGγ proteasome activators. J. Biol. Chem. 272, 25483–25492 (1997). 9325261 10.1074/jbc.272.41.25483

[59] C. Wojcik, K. Tanaka, N. Paweletz, U. Naab, S. Wilk, Proteasome activator (PA28) subunits, α, β and γ (Ki antigen) in NT2 neuronal precursor cells and HeLa S3 cells. Eur. J. Cell Biol. 77, 151–160 (1998). 9840465 10.1016/s0171-9335(98)80083-6

[60] I. Mao, J. Liu, X. Li, H. Luo, REGγ, a proteasome activator and beyond? Cell. Mol. Life Sci. 65, 3971–3980 (2008). 18679578 10.1007/s00018-008-8291-z PMC11131756

[61] L. Zannini, D. Lecis, G. Buscemi, L. Carlessi, P. Gasparini, E. Fontanella, S. Lisanti, L. Barton, D. Delia, REGγ proteasome activator is involved in the maintenance of chromosomal stability. Cell Cycle 7, 504–512 (2008). 18235248 10.4161/cc.7.4.5355

[62] A. S. Weintraub, C. H. Li, A. V. Zamudio, A. A. Sigova, N. M. Hannett, D. S. Day, B. J. Abraham, M. A. Cohen, B. Nabet, D. L. Buckley, Y. E. Guo, D. Hnisz, R. Jaenisch, J. E. Bradner, N. S. Gray, R. A. Young, YY1 is a structural regulator of enhancer-promoter loops. Cell 171, 1573–1588.e28 (2017). 29224777 10.1016/j.cell.2017.11.008 PMC5785279

[63] G. Gao, J. Wong, J. Zhang, I. Mao, J. Shravah, Y. Wu, A. Xiao, X. Li, H. Luo, Proteasome activator REGγ enhances coxsackieviral infection by facilitating p53 degradation. J. Virol. 84, 11056–11066 (2010). 20719955 10.1128/JVI.00008-10 PMC2953206

[64] I. A. Hendriks, D. Lyon, D. Su, N. H. Skotte, J. A. Daniel, L. J. Jensen, M. L. Nielsen, Site-specific characterization of endogenous SUMOylation across species and organs. Nat. Commun. 9, 2456 (2018). 29942033 10.1038/s41467-018-04957-4 PMC6018634

[65] H. S. Lee, Y. S. Lim, E. M. Park, S. H. Baek, S. B. Hwang, SUMOylation of nonstructural 5A protein regulates hepatitis C virus replication. J. Viral Hepat. 21, e108–e117 (2014). 24602294 10.1111/jvh.12241

[66] A. Akil, G. Wedeh, M. Zahid Mustafa, A. Gassama-Diagne, SUMO1 depletion prevents lipid droplet accumulation and HCV replication. Arch. Virol. 161, 141–148 (2016). 26449956 10.1007/s00705-015-2628-3

[67] J. Guo, D. Chen, X. Gao, X. Hu, Y. Zhou, C. Wu, Y. Wang, J. Chen, R. Pei, X. Chen, Protein inhibitor of activated STAT2 restricts HCV replication by modulating viral proteins degradation. Viruses 9, 285 (2017). 28973998 10.3390/v9100285 PMC5691636

[68] A. C. Bellail, J. J. Olson, C. Hao, SUMO1 modification stabilizes CDK6 protein and drives the cell cycle and glioblastoma progression. Nat. Commun. 5, 4234 (2014). 24953629 10.1038/ncomms5234 PMC4090607

[69] X.-X. Sun, Y. Chen, Y. Su, X. Wang, K. M. Chauhan, J. Liang, C. J. Daniel, R. C. Sears, M.-S. Dai, SUMO protease SENP1 deSUMOylates and stabilizes c-Myc. Proc. Natl. Acad. Sci. U.S.A. 115, 10983–10988 (2018). 30305424 10.1073/pnas.1802932115 PMC6205424

[70] Y. Chen, X.-X. Sun, R. C. Sears, M.-S. Dai, Writing and erasing MYC ubiquitination and SUMOylation. Genes Dis. 6, 359–371 (2019). 31832515 10.1016/j.gendis.2019.05.006 PMC6889025

[71] L. Fan, T. Bi, L. Wang, W. Xiao, DNA-damage tolerance through PCNA ubiquitination and sumoylation. Biochem. J. 477, 2655–2677 (2020). 32726436 10.1042/BCJ20190579

[72] R. Kumar, R. González-Prieto, Z. Xiao, M. Verlaan-de Vries, A. C. O. Vertegaal, The STUbL RNF4 regulates protein group SUMOylation by targeting the SUMO conjugation machinery. Nat. Commun. 8, 1809 (2017). 29180619 10.1038/s41467-017-01900-x PMC5703878

[73] C.-Y. Kuo, X. Li, J. M. Stark, H. M. Shih, D. K. Ann, RNF4 regulates DNA double-strand break repair in a cell cycle-dependent manner. Cell Cycle 15, 787–798 (2016). 26766492 10.1080/15384101.2016.1138184 PMC4845925

[74] M. Zimmermann, F. Lottersberger, S. B. Buonomo, A. Sfeir, T. de Lange, 53BP1 regulates DSB repair using Rif1 to control 5′ end resection. Science 339, 700–704 (2013). 23306437 10.1126/science.1231573 PMC3664841

[75] I. A. Hendriks, L. W. Treffers, M. Verlaan-de Vries, J. V. Olsen, A. C. O. Vertegaal, SUMO-2 orchestrates chromatin modifiers in response to DNA damage. Cell Rep. 10, 1778–1791 (2015). 25772364 10.1016/j.celrep.2015.02.033 PMC4514456

[76] Y. Sun, L. M. Miller Jenkins, Y. P. Su, K. C. Nitiss, J. L. Nitiss, Y. Pommier, A conserved SUMO pathway repairs topoisomerase DNA-protein cross-links by engaging ubiquitin-mediated proteasomal degradation. *Sci. Adv.* **6**, eaba6290 (2020). 10.1126/sciadv.aba6290 PMC7673754 33188014

[77] V. Lallemand-Breitenbach, M. Jeanne, S. Benhenda, R. Nasr, M. Lei, L. Peres, J. Zhou, J. Zhu, B. Raught, H. de Thé, Arsenic degrades PML or PML–RARα through a SUMO-triggered RNF4/ubiquitin-mediated pathway. Nat. Cell Biol. 10, 547–555 (2008). 18408733 10.1038/ncb1717

[78] N. Martin, K. Schwamborn, V. Schreiber, A. Werner, C. Guillier, X.-D. Zhang, O. Bischof, J.-S. Seeler, A. Dejean, PARP-1 transcriptional activity is regulated by sumoylation upon heat shock. EMBO J. 28, 3534–3548 (2009). 19779455 10.1038/emboj.2009.279 PMC2782092

[79] X. Zhao, SUMO-mediated regulation of nuclear functions and signaling processes. Mol. Cell 71, 409–418 (2018). 30075142 10.1016/j.molcel.2018.07.027 PMC6095470

[80] K. Eifler, A. C. O. Vertegaal, SUMOylation-mediated regulation of cell cycle progression and cancer. Trends Biochem. Sci. 40, 779–793 (2015). 26601932 10.1016/j.tibs.2015.09.006 PMC4874464

[81] T.-Y. Yau, W. Sander, C. Eidson, A. J. Courey, SUMO interacting motifs: Structure and function. Cell 10, 2825 (2021). 10.3390/cells10112825 PMC8616421 34831049

[82] M. Lussier-Price, X. H. Mascle, L. Cappadocia, R. Kamada, K. Sakaguchi, H. M. Wahba, J. G. Omichinski, Characterization of a C-terminal SUMO-interacting motif present in select PIAS-family proteins. Structure 28, 573–585.e5 (2020). 32348746 10.1016/j.str.2020.04.002

[83] D. Hnisz, K. Shrinivas, R. A. Young, A. K. Chakraborty, P. A. Sharp, A phase separation model for transcriptional control. Cell 169, 13–23 (2017). 28340338 10.1016/j.cell.2017.02.007 PMC5432200

[84] L. Breeden, K. Nasmyth, Regulation of the yeast HO gene. Cold Spring Harb. Symp. Quant. Biol. 50, 643–650 (1985). 3938367 10.1101/sqb.1985.050.01.078

[85] L. Guarente, Yeast promoters and lacZ fusions designed to study expression of cloned genes in yeast. Methods Enzymol. 101, 181–191 (1983). 6310321 10.1016/0076-6879(83)01013-7

[86] J. Jumper, R. Evans, A. Pritzel, T. Green, M. Figurnov, O. Ronneberger, K. Tunyasuvunakool, R. Bates, A. Žídek, A. Potapenko, A. Bridgland, C. Meyer, S. A. A. Kohl, A. J. Ballard, A. Cowie, B. Romera-Paredes, S. Nikolov, R. Jain, J. Adler, T. Back, S. Petersen, D. Reiman, E. Clancy, M. Zielinski, M. Steinegger, M. Pacholska, T. Berghammer, S. Bodenstein, D. Silver, O. Vinyals, A. W. Senior, K. Kavukcuoglu, P. Kohli, D. Hassabis, Highly accurate protein structure prediction with AlphaFold. Nature 596, 583–589 (2021). 34265844 10.1038/s41586-021-03819-2 PMC8371605

[87] C. Dominguez, R. Boelens, A. M. Bonvin, HADDOCK: A protein-protein docking approach based on biochemical or biophysical information. J. Am. Chem. Soc. 125, 1731–1737 (2003). 12580598 10.1021/ja026939x

