# Supplementary material for: SUMOylation-mediated PSME3-20S proteasomal degradation of transcription factor CP2c is crucial for cell cycle progression
Source: Sci Adv. 2023 Jan 27;9(4):eadd4969. doi: 10.1126/sciadv.add4969 (PMC9882985; doi:10.1126/sciadv.add4969)
Supplement: Supplementary file 1 — Figs. S1 to S12 Tables S1 and S2 [file sciadv.add4969_sm.pdf]

Science Advances
AAAS

Supplementary Materials for

SUMOylation-mediated PSME3-20*S* proteasomal degradation of transcription factor CP2c is crucial for cell cycle progression

Seung Han Son *et al.*

Corresponding author: Chul Geun Kim, cgkim@hanyang.ac.kr; Vladimir N. Uversky, vuversky@usf.edu

Sci. Adv. **9**, eadd4969 (2023)
DOI: 10.1126/sciadv.add4969

This PDF file includes:

Figs. S1 to S12
Tables S1 and S2

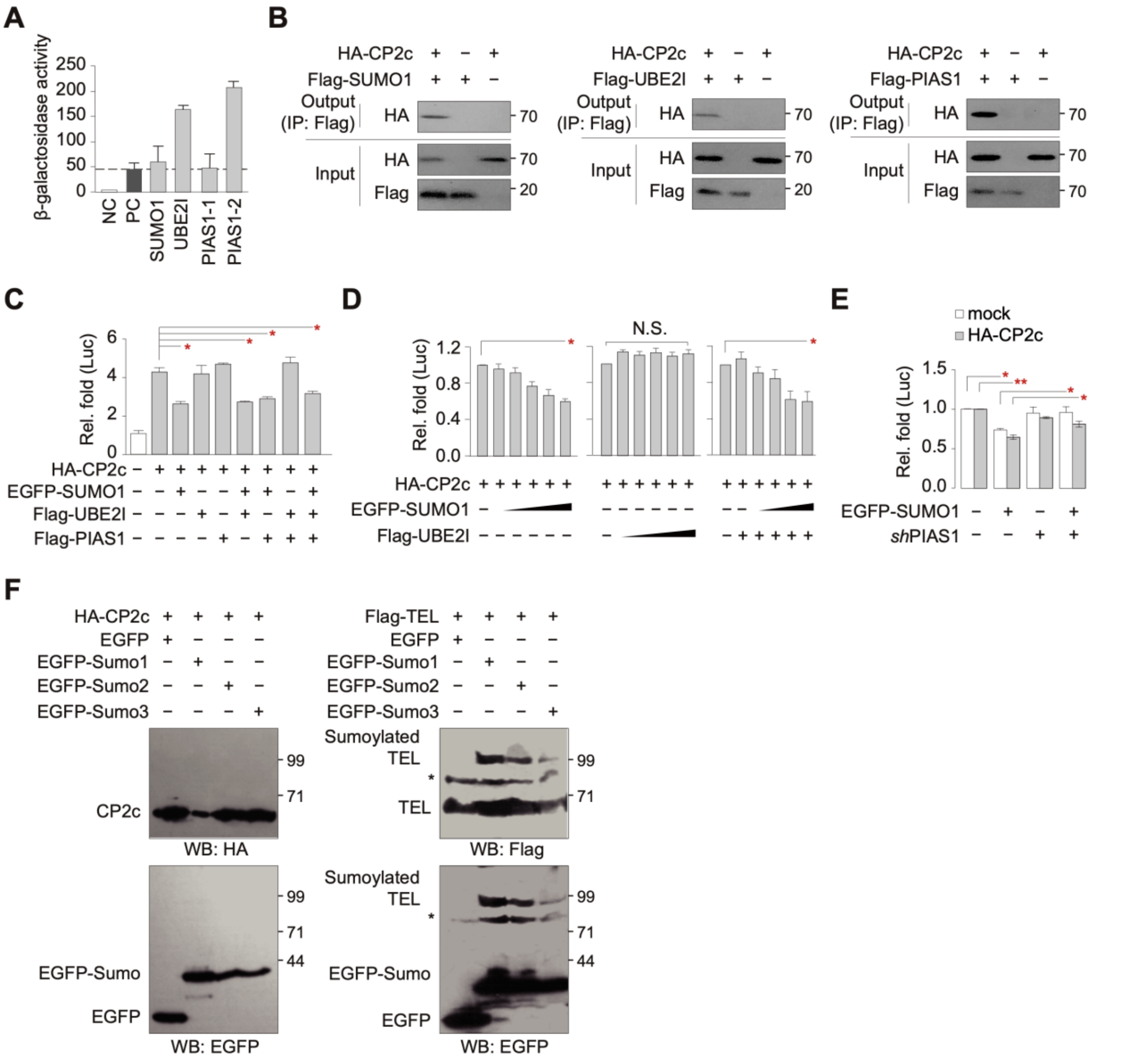

Fig. S1. SUMO1, UBE2I, and PIAS1 involvement in CP2c degradation.

(**A**) Quantification of CP2c interaction strength with SUMO1, UBE2I, and PIAS1. pLexA-CP2c (ΔN1-39) was co-transformed into the yeast strain EGY48 along with pB42AD-SUMO1, -UBE2I, or -PIAS1. The strength of protein-protein interactions was determined by the ONPG assays to measure the activity of reporter gene β-galactosidase. The pLexA-p53/pB42AD-T antigen and the pLexA-p53/pB42AD were used as a positive control (PC) and a negative control (NC), respectively. The data are shown as mean ± SEM; n = 2.

(**B**) Immunoprecipitation assays showing the direct interaction between ectopically overexpressed CP2c and SUMO1, UBE2I, or PIAS1.

(**C** – **E**) Luciferase reporter analyses showing that CP2c transcriptional activity is reduced by ectopic overexpression of SUMO1 regardless of ectopic overexpression of UBE2I or PIAS1 (C and D), but it requires UBE2I and PIAS1 (E). CP2c-tet, a synthetic promoter containing only tetrameric CP2c half-binding sites linked to the β-globin TATA box, was used as a promoter for Luc gene expression. The data are shown as mean ± SD; n = 3. *, $P < 0.05$; **, $P < 0.01$.

(**F**) CP2c is degraded by murine Sumo1, but not by Sumo2 or Sumo3. TEL1 is used as a positive control for SUMOylation. The SUMOylated CP2c is not seen due to rapid degradation of the SUMOylated CP2c unless protease inhibitor MG132 and deSUMOylation inhibitor NEM are treated. *, non-specific bands. This figure is related to Fig. 1.

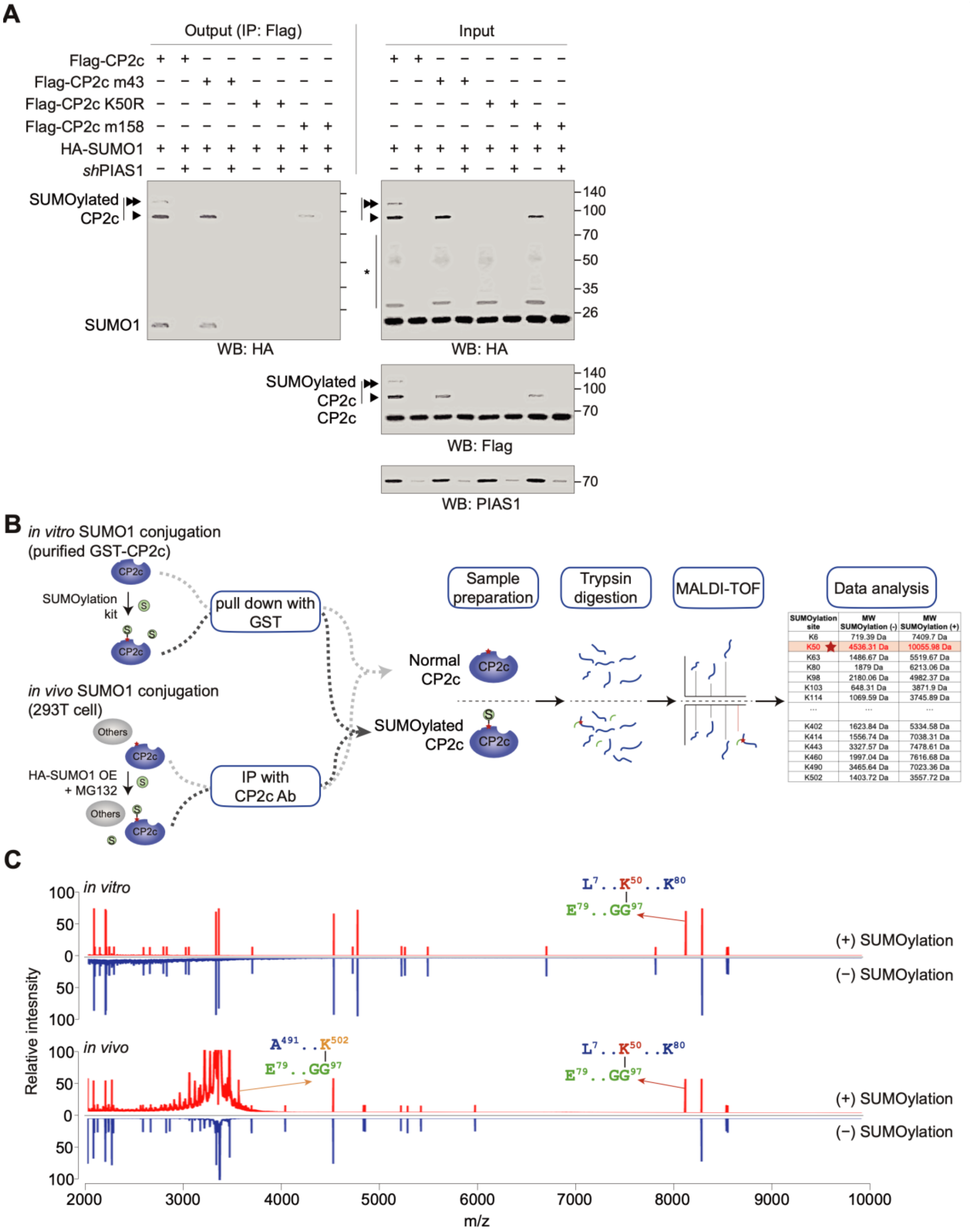

Fig. S2. Identification and characterization of the CP2c residues/regions responsible for CP2c SUMOylation and degradation.

(**A**) Both MG132 and NEM treatments were required for the detailed understanding of the PIAS1-depdendent CP2c SUMOylation phenomenon, including the identification of a SUMOylation site, SIM, and CP2c-bound SUMO1.

(**B** and **C**) Experimental scheme for the identification of SUMOylation sites by IP (or pull down)-MS analyses (B), and MS showing CP2c SUMOylation at K50 *in vitro* and *in vivo* (C). CP2c C-terminal lysine (K502) may also be SUMOylated *in vivo*. This figure is related to Fig. 2.

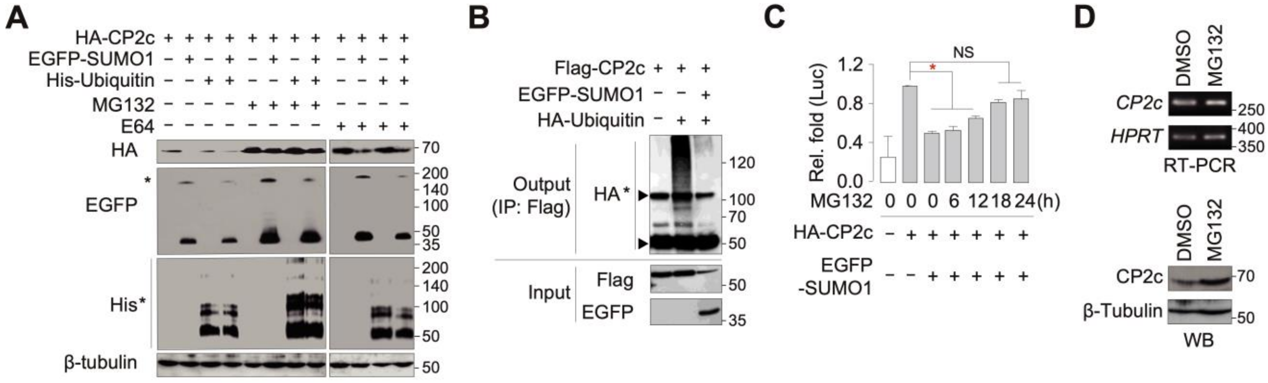

Fig. S3. The SUMO1-conjugated CP2c is degraded through the ubiquitin–independent proteasosmal pathway.

(**A**) Representative Western blots (n = 3) showing that SUMO1-mediated CP2c degradation is ablated by treatment of the proteasome inhibitor MG132, but not by the lysosome inhibitor E64. *, other SUMOylated or ubiquitinated proteins.

(**B**) Representative Co-IPs (n = 3) showing SUMO1 mediated CP2c degradation is ubiquitin independent. *, non-specific bands.

(**C**) Luciferase reporter analyses showing that SUMO1-dependent reduction of the CP2c transcriptional activity is protected by MG132 (10 μM) in a treatment-time-dependent manner. Error bars indicate SEM from three independent transfections. *, $P < 0.05$.

(**D**) Representative RT-PCR and Western analyses (n = 2) showing the MG132 (50 μM) treatment blocks the SUMO1-mediated CP2c degradation, without affecting the *CP2c* mRNA level.

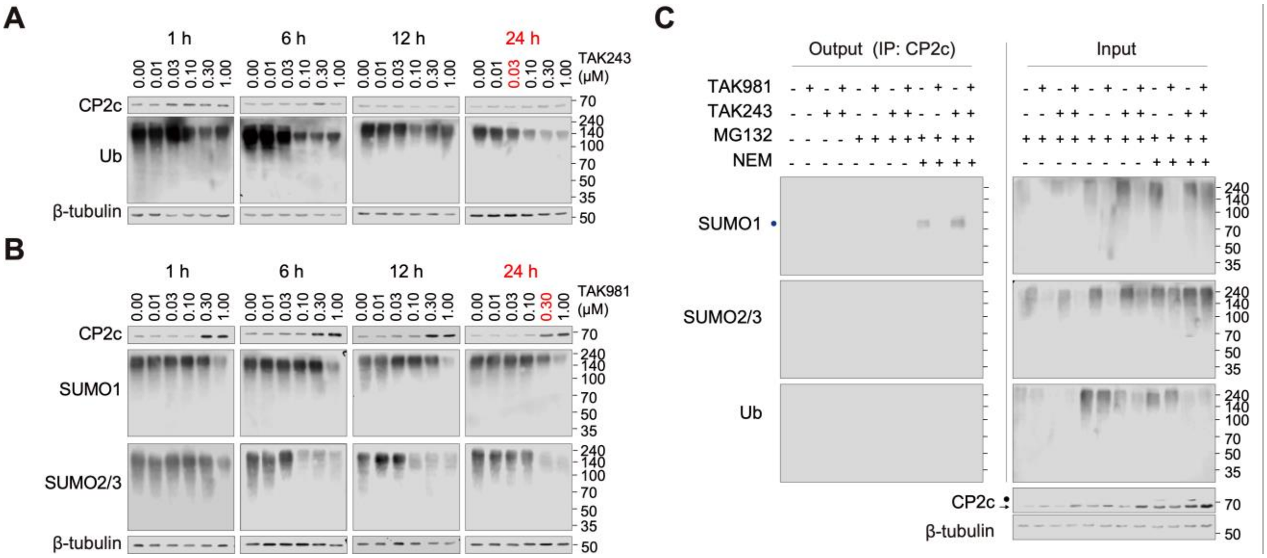

Fig. S4. CP2c degradation occurs in a SUMOylation-, but not a ubiquitin–dependent manner. (**A** and **B**) Representative western blot analyses (n = 2) showing dose response and time course of the TAK-243-independent (A) and TAK981-dependent (B) CP2c degradation in MDA-MB-231 cells. β-tubulin was used as a loading control. From these data, cells were treated with 0.03 μM TAK243 and 0.30 μM TAK981 for 24 h to inhibit ubiquitination and SUMOylation of the endogenous substrates in all of the subsequent experiments.

(**C**) Representative Co-IPs (n = 3) demonstrating that SUMO1-mediated SUMOylation, but not SUMO2/3-mediated SUMOylation or ubiquitination, of endogenous CP2c is involved in the CP2c degradation *in vivo*. A colored dot represents SUMOylated CP2c.

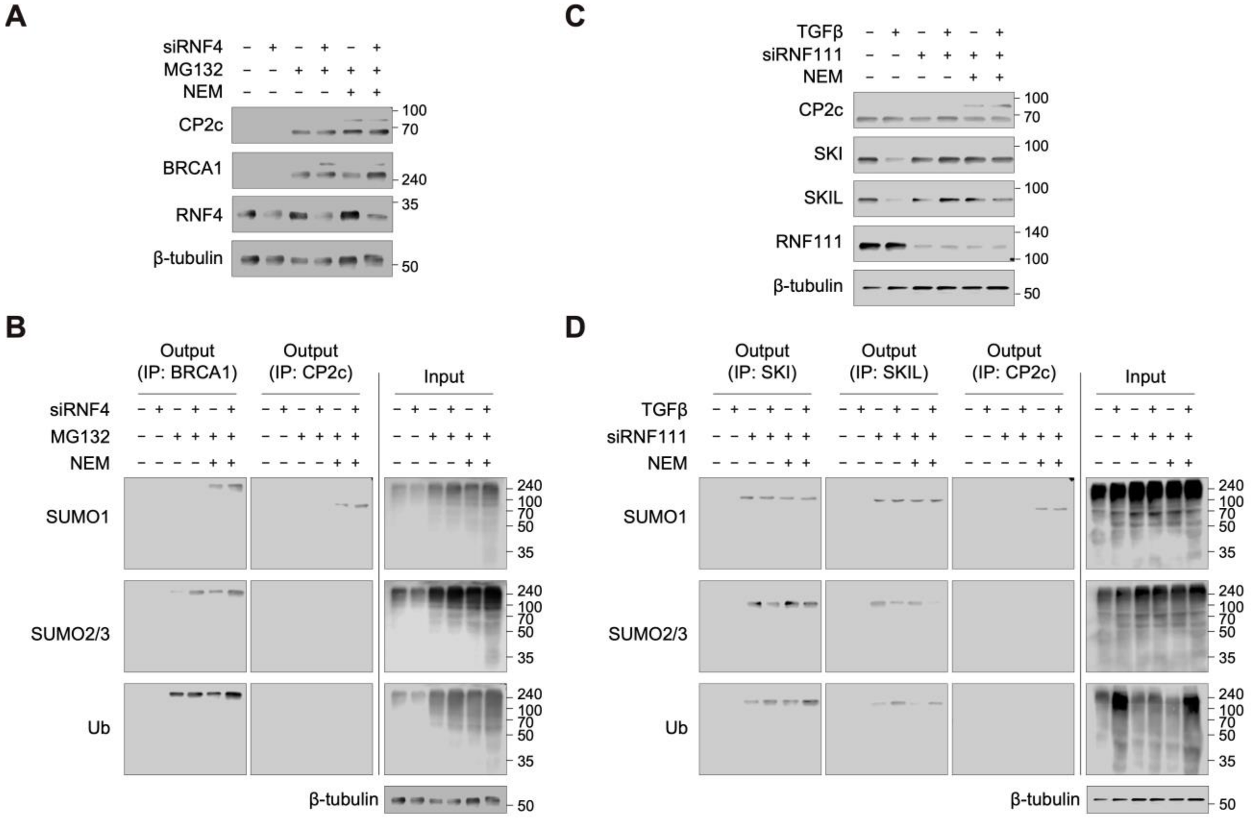

Fig. S5. The SUMO1-conjugated CP2c is degraded in a STUbLs–independent manner.
(**A** and **B**) CP2c is not degraded by RNF4 STUbL. BRCA1 was used as a positive RNF4 substrate (*41*). Representative (n = 3) Western blots (A) and Co-IPs (B) were performed to see protein expression levels or changes of SUMOylation and/or ubiquitination profiles of BRCA1 and CP2c in MDA-MB-231 cells by transient transfection of siRNF4, respectively.
(**C** and **D**) CP2c is not degraded by RNF111 STUbL. SKI and SKIL were used as positive RNF111 substrates in TGFβ-treated cells (*48*). Representative (n = 3) Western blots (C) and Co-IPs (D) were performed to see protein expression levels or changes of SUMOylation and/or ubiquitination profiles of control substrates and CP2c in 293T cells by transient transfection of siRNF111, respectively.

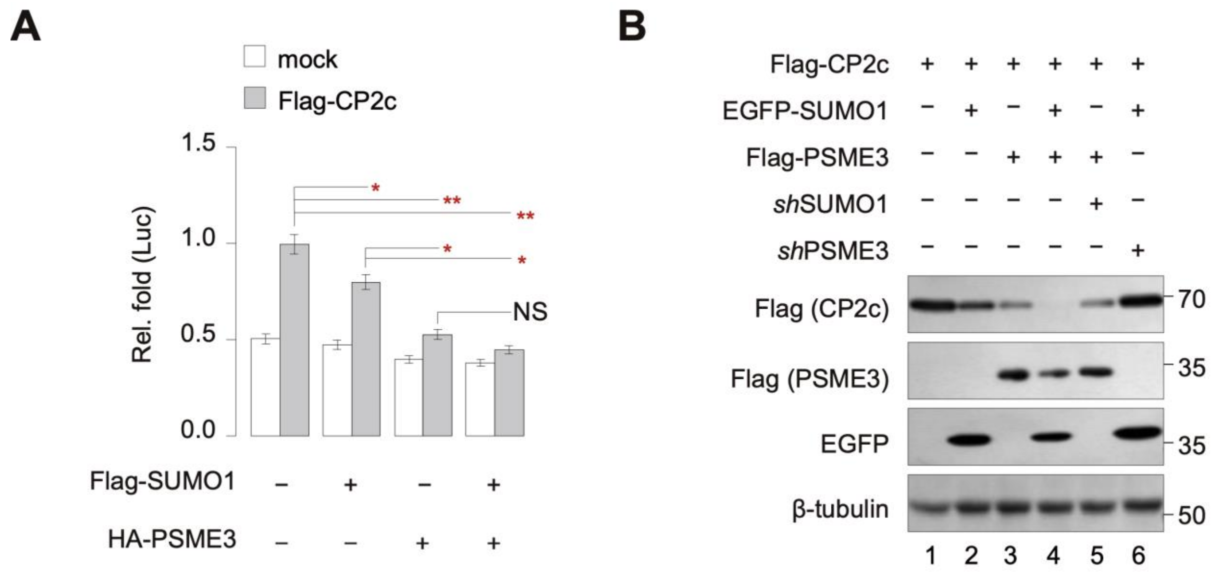

Fig. S6. Both SUMO1 and PSME3 are involved in the CP2c degradation.

(**A**) Luciferase reporter analysis showing that the CP2c transcriptional activity is decreased by SUMO1 or PSME3. Error bars indicate SEM from three independent transfections. *; $P < 0.05$, **; $P < 0.01$.

(**B**) Representative Western blots (n = 3) showing the dramatically reduced CP2c protein level in the SUMO1 and PSME3 co-transfected cells.

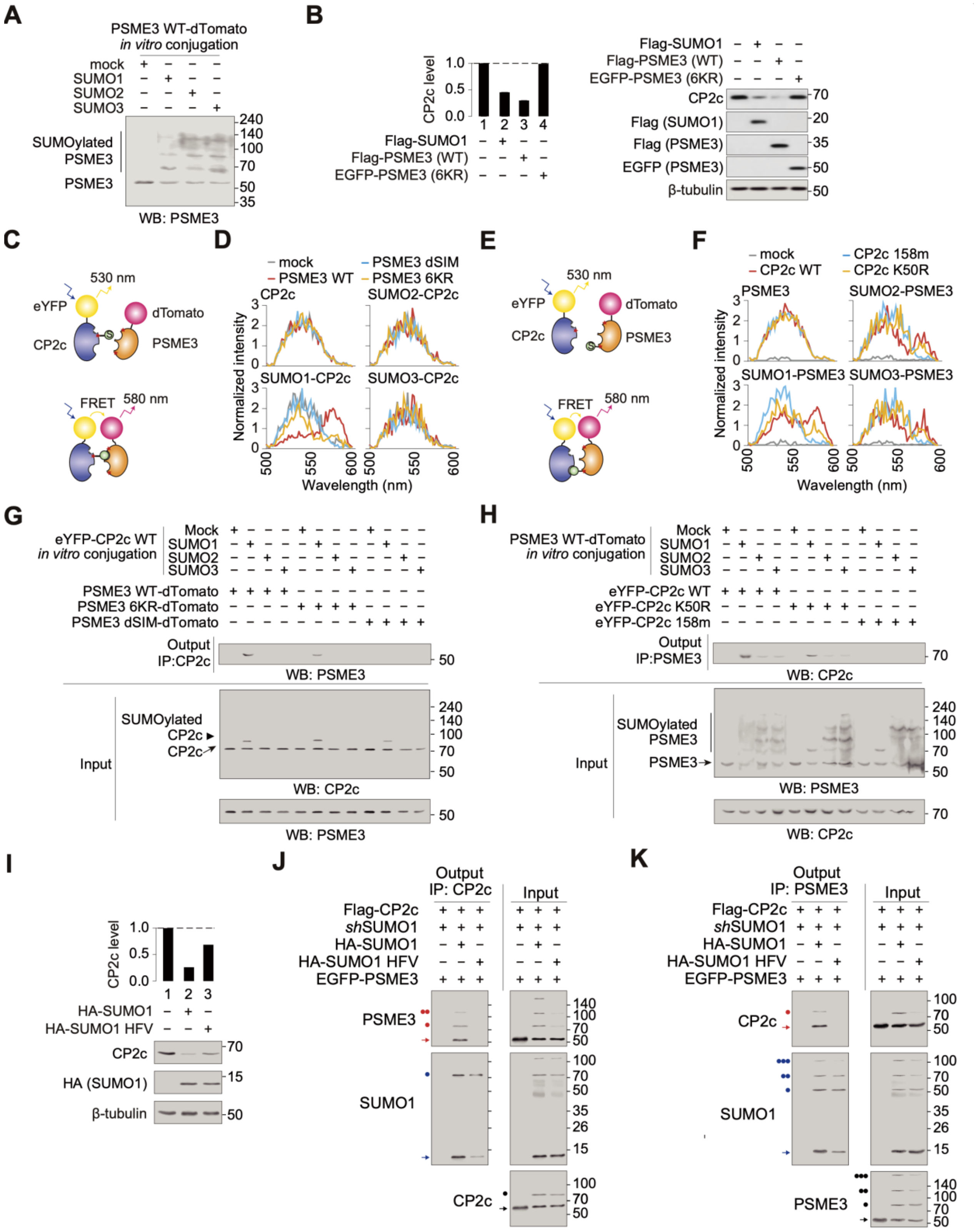

Fig. S7. PSME3-mediated CP2c degradation occurs by either interaction of the SUMOylated CP2c with the PSME3 SIMs or vice versa.

(**A**) Representative Western blot (n = 2) showing *in vitro* PSME3 SUMOylation by SUMO1, SUMO2, or SUMO3.

(**B**) Representative Western blot (n = 2) showing the PSME3 6KR mutant effect on CP2c protein degradation.

(**C** and **D**) Schematic drawing of FRET assays in between the SUMOylated eYFP-CP2c and the dTomato-PSME3 (C), and FRET graphs showing the distribution of *in vitro* FRET states between the eYFP-CP2c WT and the SUMO-conjugated PSME3 WT or mutants (D).
(**E** and **F**) Schematic drawing of FRET assays in between the eYFP-CP2c and the SUMOylated dTomato-PSME3 (E), and FRET graphs showing the distribution of *in vitro* FRET states between the eYFP-CP2c WT or mutants and the SUMO-conjugated PSME3 (F). Averaged FRET dynamics were obtained by 3 individual experiments.
(**G** and **H**) Representative Co-IPs (n = 2) showing interactions between the SUMOylated CP2c and the PSME3 WT or mutants (G), and between the SUMOylated PSME3 and the CP2c WT or mutants (H).
(**I**) Representative Western blot (n = 2) showing the SUMO1 HFV mutant effect on CP2c protein degradation.
(**J** and **K**) representative Co-IP assays (n = 2) showing that CP2c and PSME3 recognize each other through the direct CP2c-SUMO1-PSME3 interaction *in vivo*. Epitope-tagged CP2c and PSME3 were transiently transfected into cells along with various combinations of *sh*SUMO1, WT SUMO1, and SUMO1 HFV (H35A/F36A/V38A) mutant. Co-IPs analyses were done by IP with CP2c (J) or PSME3 (K).

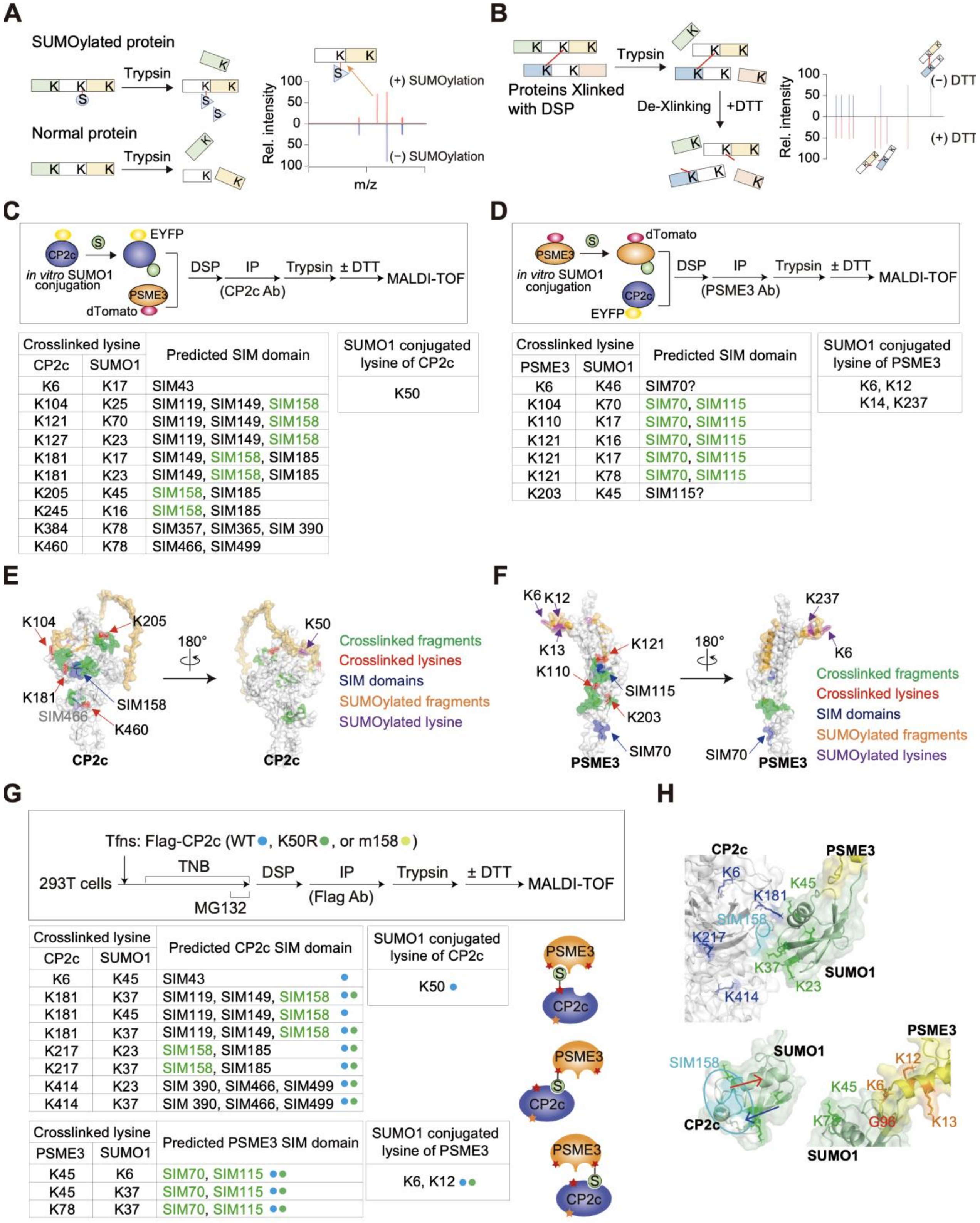

Fig. S8. Validation of interactions between CP2c and PSME3 through mutual SUMO-SIM binding.

(**A** and **B**) Experimental schemes for identifying peptide fragments containing a SUMOylation site by MS (A) and peptide fragments involved in protein-protein interaction by DSP crosslinking-MS (B), respectively.

(**C** and **D**) Identification of CP2c SUMOylation sites and SIMs responsible for the PSME3 interaction (C), and PSME3 SUMOylation sites and SIMs responsible for the CP2c interaction (D). Experimental schemes are shown at the top.
(**E** and **F**) Three-dimensional structural models of CP2c (E) and PSME3 (F), highlighting the amino acd residues or peptide regions involved in the crosslinking, SUMOylation, or SIM function.
(**G**) CP2c-PSME3 interaction involves mutual binding between the SUMOylation sites and SIMs. A DSP crosslinking-IP-WB protocol (top) was employed in the tests of interaction between the WT PSME3 and the WT or mutant CP2c.
(**H**) Structural models showing the SUMOylation site/SIM binding interfaces in the CP2c-PSME3 interaction. This figure is related to Fig. 4.

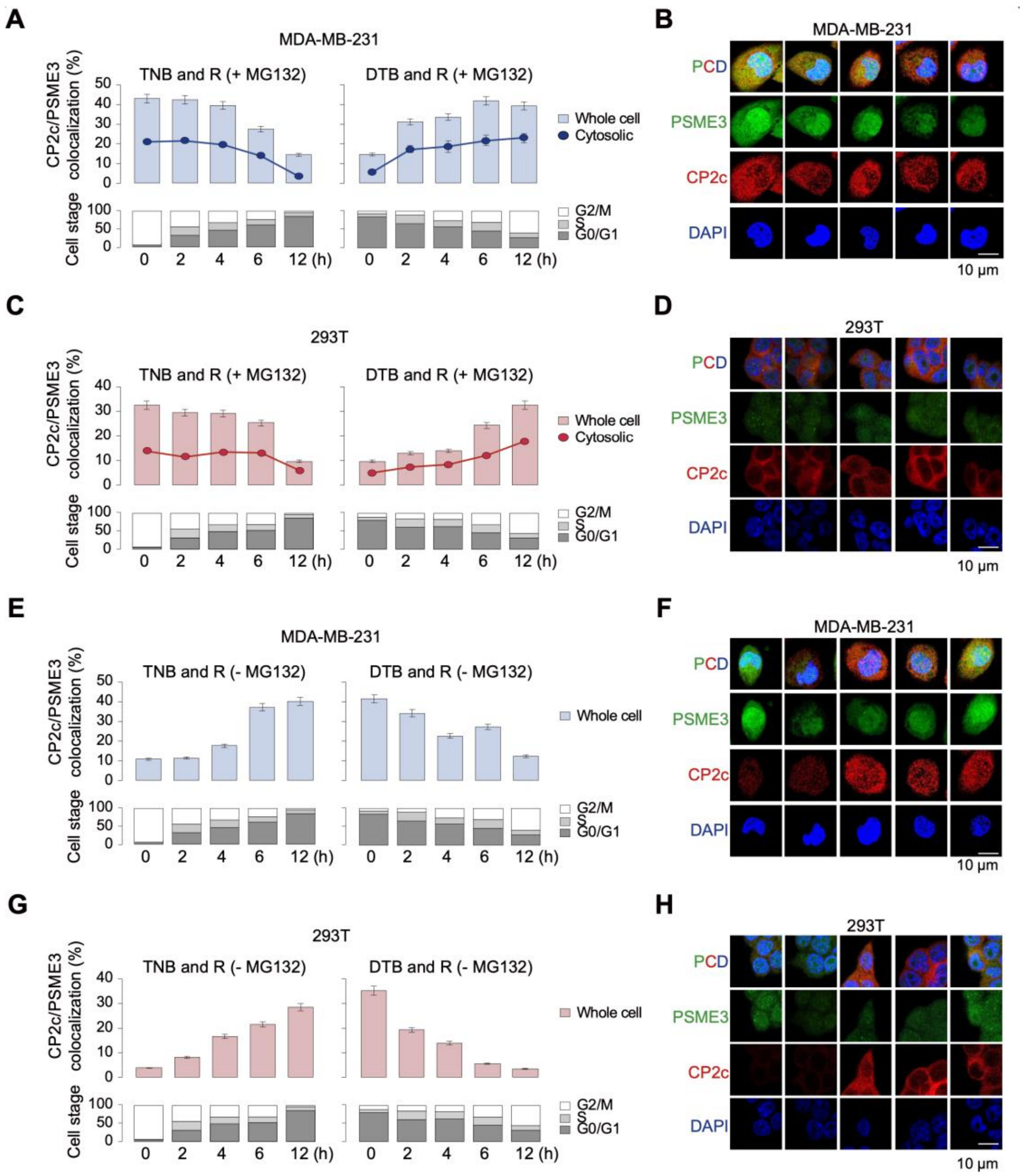

Fig. S9. SUMO1/PSME3-mediated CP2c degradation ocurrs in the cytosol of the G2/M /G1 phase cells.

(**A** to **D**) CP2c and PSME3 colocalize at the G2/M/G1 phase of the cell cycle. The percentage of CP2c/PSME3 colocalization in whole cells or in the cytosol was quantified by immunofluorescence microscopic image analyses of MDA-MB-231 and 293T cells (A and C) collected at each time points after release from TNB or DTB. MG132 (10 μM) was added to cells for 6 h before releasing the cell cycle arrest to enhance colocalization signals by preventing CP2c degradation. The percentage of cell populations at each time point are shown at the bottom. Representative confocal images are shown in B and D. The data are shown as mean ± SD; n = 2.

(**E** to **H**) CP2c degradation occurs at the G2/M/G1 phase of the cell cycle, reducing the amounts of the CP2c and PSME3 colocalization. The percentage of the CP2c/PSME3 colocalization in whole cells was quantified by the immunofluorescence microscopic image analyses of MDA-MB-231 and 293T cells (E and G) collected at each time points after the release from TNB or DTB in the absence of MG132 treatment. The percentage of cell populations at each time point are shown at the bottom. Representative confocal images are shown in F and H. The data are shown as mean ± SD; n = 2.

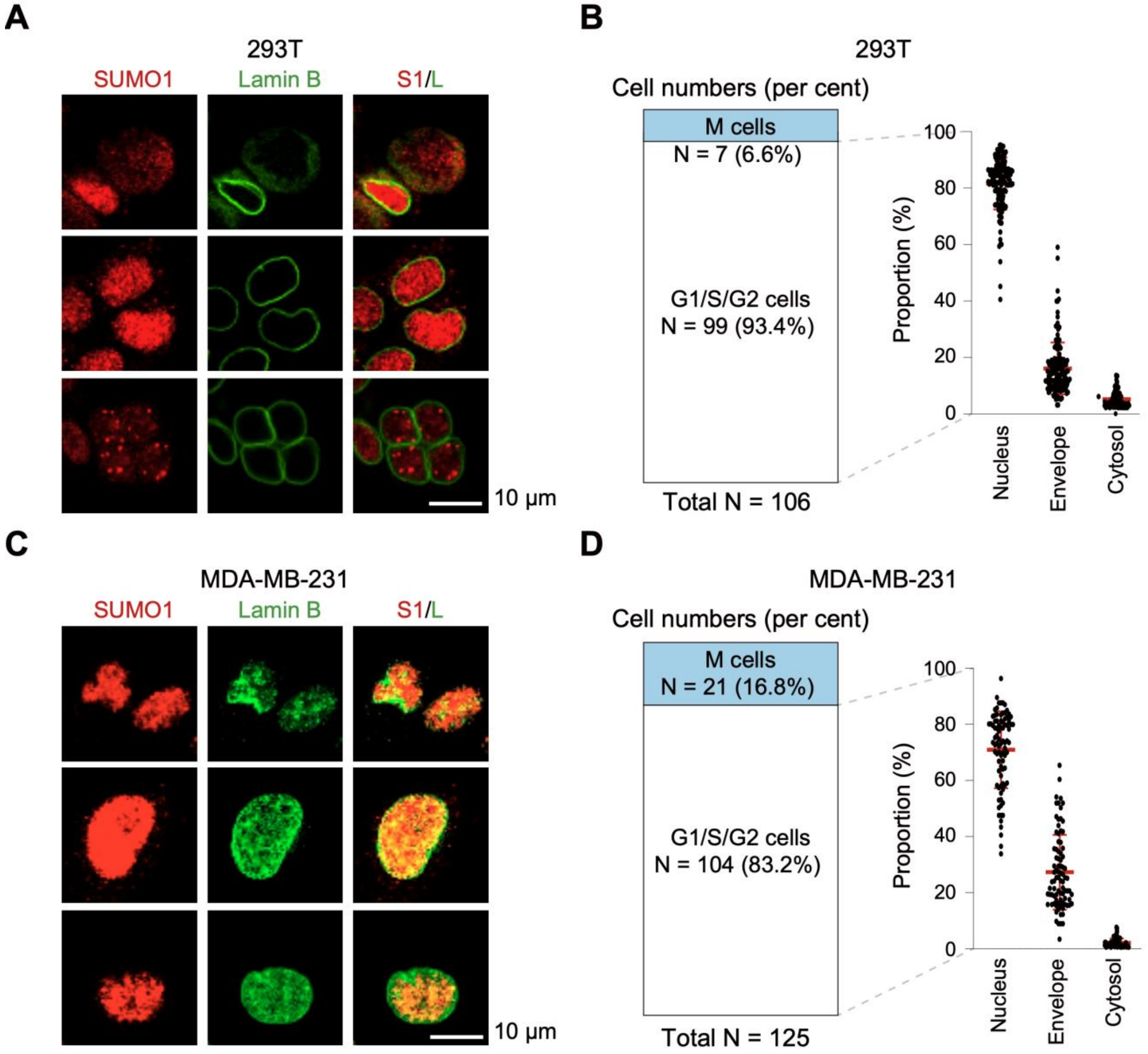

Fig. S10. **Cytoplasmic SUMO1 appears in some cells entering into or exiting from mitosis.** Representative confocal images immunofluorescence showing cellular distribution of SUMO1 in 293T (**A**) and MDA-MB-231(**C**) cells. Lamin B staining was included to distinguish the nucleus and cytoplasm of cells. Cellular distribution of SUMO1 scored in more than 100 cells is shown in **B** and **D**. It is important to note that we can no longer distinguish cytoplasm from nucleus in mitotic cells because of nuclear envelop breakdown, and therefore we excluded those mitotic cells from the counting of cells for the cellular distribution of SUMO1.

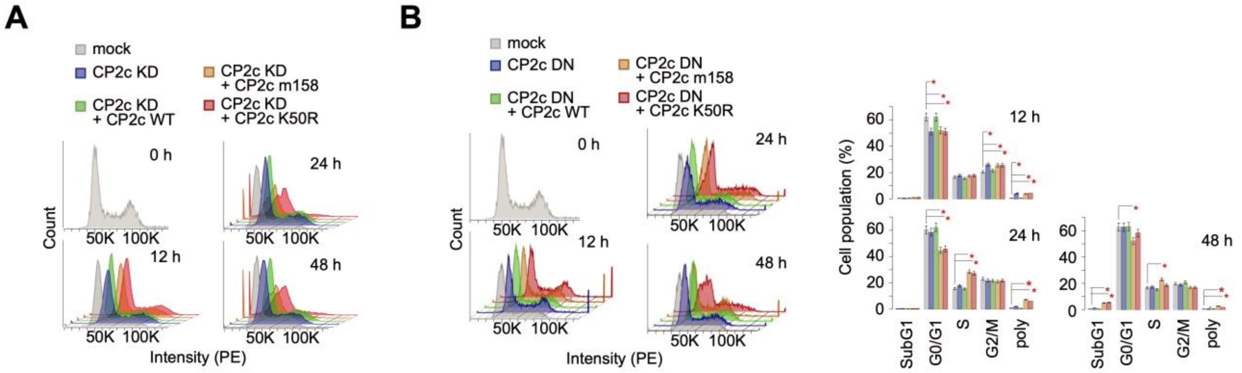

Fig. S11. SUMO1/PSME3-mediated CP2c degradation at the cytosol of the G2/M /G1 phase cells is crucial for the proper cell cycle progression.
(**A** and **B**) Flow cytometry cell cycle analyses showing importance of the SUMO1/PSME3 proteasome axis-dependent CP2c degradation for the proper cell cycle progression. Flow cytometry was employed to analyze cell cycle profiles in the cells with CP2c mutation in the SUMO binding site (K50R) or SIM (m158) at 48 h after transfection. Endogenous CP2c expression was suppressed in 293T cells by the transfection of the CP2c shRNA (A) or CP2c dominant negative (*62*) (B) expression vector. n = 2, *, $P < 0.05$. This figure is related to Fig. 5.

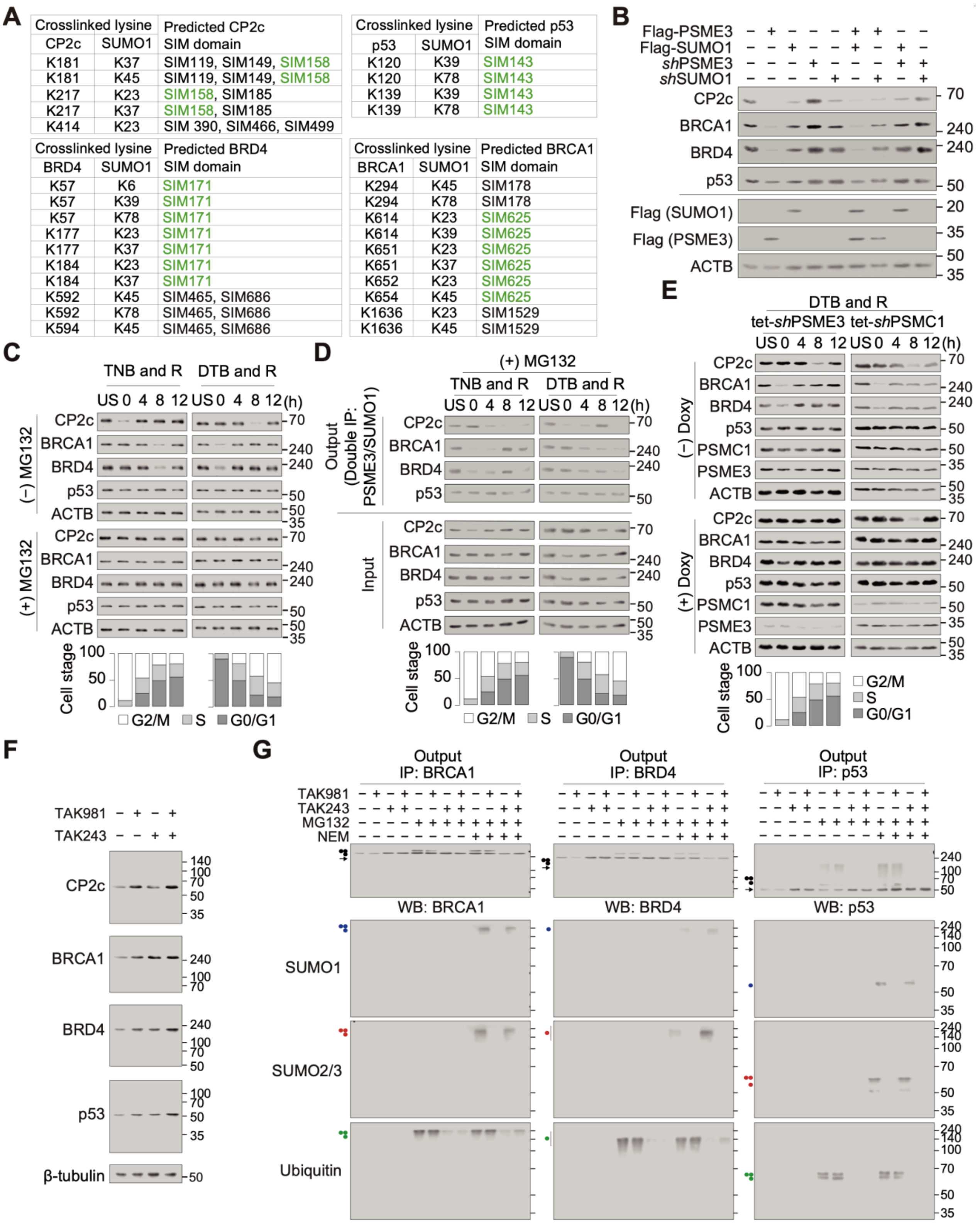

Crosslinked lysine		Predicted CP2c
CP2c	SUMO1	SIM domain
K181	K37	SIM119, SIM149, SIM158
K181	K45	SIM119, SIM149, SIM158
K217	K23	SIM158, SIM185
K217	K37	SIM158, SIM185
K414	K23	SIM 390, SIM466, SIM499

Crosslinked lysine		Predicted p53
p53	SUMO1	SIM domain
K120	K39	SIM143
K120	K78	SIM143
K139	K39	SIM143
K139	K78	SIM143

Crosslinked lysine		Predicted BRD4
BRD4	SUMO1	SIM domain
K57	K6	SIM171
K57	K39	SIM171
K57	K78	SIM171
K177	K23	SIM171
K177	K37	SIM171
K184	K23	SIM171
K184	K37	SIM171
K592	K45	SIM465, SIM686
K592	K78	SIM465, SIM686
K594	K45	SIM465, SIM686

Crosslinked lysine		Predicted BRCA1
BRCA1	SUMO1	SIM domain
K294	K45	SIM178
K294	K78	SIM178
K614	K23	SIM625
K614	K39	SIM625
K651	K23	SIM625
K651	K37	SIM625
K652	K23	SIM625
K654	K45	SIM625
K1636	K23	SIM1529
K1636	K45	SIM1529

Fig. S12. The SUMO1/PSME3 proteasome system functions in cell cycle dependent degradation of some other nuclear factors.

(**A**) Identification of SIM-containing peptide fragments responsible for the SUMOylated PSME3 interaction (see Fig. 6B for the identification of candidate protein list).

(**B**) Representative Western blots (n = 2) showing the SUMO1 and/or PSME3-dependent modulation of protein degradation. 293T cells were transfected with various SUMO1 and PSME3

constructs, alone or in combination. Protein expression levels were quantified by Western blotting at 48 h after transfection.

(**C**) Representative Western blots (n = 2) showing cell cycle stage-dependent protein levels in cells prepared by a TNB & R or a DTB & R protocol in the presence or absence of MG132. Cell cycle profiles at specific time point are shown at the bottom.

(**D**) Representative Co-IPs (n = 2) showing cell cycle stage-dependent protein interactions with SUMO1 and/or PSME3. Cells were prepared by the TNB & R or DTB & R protocol in the presence of MG132.

(**E**) Representative Western blots (n = 2) showing protein levels as a whole or at specific cell cycle stage in cells where PSME3 or PSMC1 expression is down regulated. Cells were prepared by the TNB & R or DTB & R protocol in the presence or absence of MG132.

(**F** and **G**) Representive Western blots (n = 2) showing cellular protein levels (F) and Co-IPs demonstrating the causative degradation pathways of endogenous proteins (G) in cells treated with specific inhibitors, including those for SUMOylation and/or ubiquitination system. Colored dots represent SUMOylated or ubiquitinated factors, respectively. These Western blots and Co-IPs were obtained from the same input samples shown in fig. S4C.

Supplementary table 1. Oligonucleotide lists

Name	Forward	Reverse
	**For vector construction**	
CP2c (GST, Flag tag)	5'-GGA ATT CAT GGC CTG GGC TCT GAA G-3'	5'-CTC GAG CTT GAG AAT GAC ATG ATA-3'
CP2c (in vitro FRET)	5'-GGT ACC ATG GCC TGG GCT CTG AA-3'	5'-AAG CTT CTA CTT CAG TAT GAT ATG ATA GC-3'
CP2c (in vivo FRET)	5'-GTA AGA TCT ATG GCC TGG GCT CTG AAG-3'	5'-CCC GAA TTC CTT GAG AAT GAC ATG ATA-3'
SUMO1 ∆GG	5'-AA GCT TCG ATG TCT GAC CAG-3'	5'-GGA TCC CGT TTG TTC CTG-3'
PSME3 (in vitro FRET)	5'-GGA TCC CAT GGC CTC GTT GCT GAA G-3'	5'-GAA TTC GTA CAG AGT CTC TGC ATT G-3'
PSME3 (in vivo FRET)	5'-GTA CTC GAG ATG GCC TCG TTG CTG AAG-3'	5'-CTA ACT AGT GCG TAC AGA GTC TCT GCA TT-3'
CP2c SIM 43	5'-CTT ATA GTA TGA GTG ATA AAC GCG CAA GGC CCA TTT TTA AGC AAG AAG-3'	5'-CTT CTT GCT TAA AAA TGG GCC TTG CGC GTT TAT CAC TCA TAC TAT AAG-3'
CP2c SIM 158	5'-GAT ATC CCA ATG TCT AAG GGT AAA ATT GAT CCC AGG GCC AAT CC-3'	5'-GGA TTG GCC CTG GGA TCA ATT TTA CCC TTA GAC ATT GGG ATA TC-3'
CP2c SIM 357	5'-CTT CTC AGG GGC AGA TAG ACG GAA ACG AAC TAG AGA CGA CGT GAT C-3'	5'-GAT CAC GTC GTC TCT AGT TCG TTT CCG TCT ATC TGC CCC TGA GAA G-3'
CP2c SIM365	5'-CTA ACT AGA GAC GAC AAG CGC CAA CGC TGC GGC CCT GCA GAT G-3'	5'-CAT CTG CAG GGC CGC AGA GTT GGC GCT TGT CGT CTC TAG TTA G-3'
CP2c K50R	5'-CGC ATT GCC CAT TTT TAG GCA AGA AGA GTC AAG-3'	5'-CTT GAC TCT TCT TGC CTA AAA ATG GGC AAT GCG-3'
PSME3 SIM70	5'-GTC CCT GAC CCC ATT CGT CGC ACC AAT AGC CAT GAT G-3'	5'-CAT CAT GGC TAT TGG TGC GAC GAA TGG GGT CAG GGA C-3'
PSME3 SIM115	5'-GAA AAG CAA CCA GCA GCG GAA GGA CAT TAT TGA GAA AG-3'	5'-CTT TCT CAA TAA TGT CCT TCC GCT GCT GGT TGC TTT TC-3'
SUMO1 H35A/F36A	5'-GGA TAG CAG TGA GAT TGC CGC CAA AGT GAA AAT GAC AAC-3'	5'-GTT GTC ATT TTC ACT TTG GCG GCA ATC TCA CTG CTA TCC-3'
SUMO1 V38A	5'-GCA GTG AGA TTC ACT TCA AAG CGA AAA TGA CAA CAC ATC-3'	5'-GAT GTG TTG TCA TTT TCG CTT TGA AGT GAA TCT CAC TGC-3'
*sh*mSUMO1	5'-GAT CCC CCG ATA AGA AGG AAG GAG AAT TCA AGA GAT TCT CCT TCC TTC TTA TCG TTT TTG GAA A-3'	5'-AGC TTT TCC AAA AAC GAT AAG AAG GAA GGA GAA TCT CTT GAA TTC TCC TTC CTT CTT ATC GGG G-3'
*sh*SUMO1	5'-GAT CCC CGG ATA AGA AGG AAG GTG AAT TCA AGA GAT TCA CCT TCC TTC TTA TCC TTT TTG GAA A-3'	5'-AGC TTT TCC AAA AAG GAT AAG AAG GAA GGT GAA TCT CTT GAA TTC ACC TTC CTT CTT ATC CGG G-3'
*sh*UBE2I	5'-GAT CCC CGA ACT TCT AAA TGA ACC AAT TCA AGA GAT TGG TTC ATT TAG AAG TTC TTT TTA -3'	5'-GGG CTT GAA GAT TTA CTT GGT TAA GTT CTC TAA CCA AGT AAA TCT TCA AGA AAA ATT CGA -3'
*sh*PIAS1	5'-GAT CCG GAA TAA GGA ATC CGG ATC CTT CCT GTC AGA GAT CCG GAT TCC TTA TTC CTT TTT G-3'	5'-AAT TCA AAA AGG AAT AAG GAA TCC GGA TCT CTG ACA GGA AGG ATC CGG ATT CCT TAT TCC G-3'

*sh*PSME3	5'-GAT CCC CGA AGC CTT CCA AGG AAC CAT TCA AGA GAT GGT TCC TTG GAA GGC TTC TTT TTG GAA A-3'	5'-AGC TTT TCC AAA AAG AAG CCT TCC AAG GAA CCA TCT CTT GAA TGG TTC CTT GGA AGG CTT CGG G-3'
*sh*PSMC1	5'-GAT CCC CGA TGA TAA TCA CGC CAT TGT TCA AGA GAC AAT GGC GTG ATT ATC ATC TTT TTG GAA A-3'	5'-AGC TTT TCC AAA AAG ATG ATA ATC ACG CCA TTG TCT CTT GAA CAA TGG CGT GAT TAT CAT CGG G-3'
**For RT-PCR**		
CP2c	5'-GCA CTC GGC CAG CTG CCA GAT C-3'	5'-TGG AGG GGG TGG TTC TGG CT-3'
HPRT	5'-GCT GGT GAA AAG GAC CTC T-3'	5'-CAC AGG ACT AGA ACA CCT GC-3'
**For siRNA transfection**		
*si*RNF4	Santa Cruz, Cat # sc-38236	
siRNF111	5'-GGA GAU GAC UCA AGG AGA A-3'	5'-UUC UCC UUG AGU CAU CUC C-3'

Supplementary table 2. Antibody lists and sources

Antibodies	Source	Catalog number
rabbit CP2c	Abcam	ab42973-100
mouse CP2c	BD bioscience	610818
mouse SUMO1	Thermo scientific	33-2400
mouse SUMO1	Santa Cruz	sc-5308
rabbit SUMO1	Santa Cruz	sc-9060
mouse SUMO2/3	Abcam	ab81371
rabbit SUMO2/3	Abcam	ab22654
goat UBE2I	Santa Cruz	sc-5229
rabbit PIAS1	Santa Cruz	sc-14016
rabbit PIAS1	Abcam	ab32219-100
mouse PIAS1	Santa Cruz	sc-365127
mouse PSME3	Santa Cruz	sc-136025
rabbit PSME3	ZYMED	38-3800
mouse BRCA1	Santa Cruz	sc-642
mouse BRD4	Santa Cruz	sc-518021
mouse p53	Santa Cruz	sc-126
mouse RNF4	Santa Cruz	sc-517643
rabbit RNF111	Abnova	H00054778-M05
mouse SKI	Santa Cruz	sc-33693
rabbit SKIL	Proteintech	178101-1AP
mouse β-tubulin	Santa Cruz	sc-55529
goat ACTB	Santa Cruz	sc-1616
rabbit Lamin B	Abcam	ab16048
mouse HA	Abcam	49969
rabbit HA	Abcam	ab137838
rabbit His	ABM	G020-ABM
mouse GST	Santa Cruz	sc-136
rabbit-FLAG	Abcam	ab2493
mouse-FALG-M2	Abcam	ab49763

FLAG-M2 bead	Sigma-Aldrich	A2220
mouse EGFP	Santa Cruz	sc-9996
rabbit EGFP	Abcam	ab6556
anti-rabbit IgG-HRP	Ab frontier	LF-SA8002
anti-mouse IgG-HRP	Thermo Fisher	31430
anti-goat IgG-HRP	Invitrogen	811260
IP Detection Reagent (HRP)	Abcam	ab131366
anti-mouse IgG-Cy3	Vectorlabs	CY-2300
anti-rabbit IgG-FITC	Vectorlabs	FI-1000
anti-goat IgG-FITC	Vectorlabs	CY-2500-1
